# Supplementary material for: A Self-Immolative Linker for the pH-Responsive Release of Amides
Source: Molecules. 2023 Mar 7;28(6):2445. doi: 10.3390/molecules28062445 (PMC10060004; doi:10.3390/molecules28062445)
Supplement: Supplementary file 1 [file molecules-28-02445-s001.zip › molecules-2227036-supplementary.pdf]

A Self-Immolative Linker for the pH-Responsive Release of Amides

Agnese Petrini, Giovanni Ievoli, Francesca Migliorini, Maurizio Taddei * and Sofia Siciliano

General experimental procedures, materials and instruments S2

^{1}H and ^{13}C NMR Spectra (Figure S1-S16) S3

Figure S17: HPLC-MS of pure compounds **15-18** S19

Figure S18-S19: Hydrolysis of compound **15** at pH 5.5. S20

Figure S20-S21: Hydrolysis of compound **16** at pH 5.5. S21

Figure S22-S23: Hydrolysis of compound **17** at pH 5.5. S22

Figure S24-S25: Hydrolysis of compound **18** at pH 5.5. S23

Figure S26: HPLC of pure compounds **8a**, **8b**, **10.** S24

Figure S27: Behaviour of compound **8a** after 48 h at different pH values S25

Figure S28: Behaviour of compound **8b** after 48 h at different pH values S26

Figure S29: Behaviour of compound **10** after 48 h at different pH values S27

Figure S30: HPLC spectra of extracted products after 36 h in human plasma S28

General experimental procedures, materials and instruments

All reagents were used as purchased from commercial suppliers without further purification. The reactions were carried out in oven dried vessels. Solvents were dried and purified by conventional methods prior use or, if available, purchased in anhydrous form. Flash column chromatography was performed with Merck silica gel 60, 0.040-0.063 mm (230-400 mesh). MPLC Isolera Prime Biotage on highly resistant PP cartridges Normal Phase silica gel NP 40 – 63 μm particle size and 60 Å pore size (Si60) withstand a maximum pressure of 10 bar (145 psi) column with petroleum ether (eluent A) and Ethyl Acetate (Eluent B) as mobile phase. Merck aluminium backed plates pre-coated with silica gel 60 (UV254) were used for analytical thin layer chromatography and were visualized by staining with a $KMnO_4$ solution. NMR spectra were recorded at 25 °C or at 37 °C and 400 MHz for ^1H and 101 MHz for ^{13}C Brucker Advance NMR spectrometers. The solvent is specified for each spectrum. Splitting patterns are designated as s, singlet; d, doublet; t, triplet; q, quartet; m, multiplet; bs, broad singlet. Chemical shifts (δ) are given in ppm relative to the resonance of their respective residual solvent peaks. High- and low-resolution mass spectroscopy analyses were recorded by electrospray ionization with a mass spectrometer Q-exactive Plus. HPLC analyses were performed on the chromatographic system JASCO LC-Net II/ ADC connected with UV detector (254 nm) using an Intersil ODS-3V C18 column (5 μm, 4.6 x 250mm), flow 0.8 mL/min, eluent A/B: H_2O/MeCN Gradient 20% B to 80% B in 45 minutes, 15 minutes at 95 % B. HPLC/MS analyses were performed on a InfinityLab LC/MSD iQ apparatus. Method: column: InfinityLab PoroShell 120 EC-C18 4.6 x 100 mm x 2.7μm; flow: 1.5 mL/min; eluent A/B: H_2O/MeCN, gradient: 5% B to 95% B in 10 minutes, 4 minutes at 95 % B and 3 minutes of re-equilibration; detection: 254 nm and 210 nm.

^{1}H and ^{13}C NMR Spectra (Figure S1-S16)

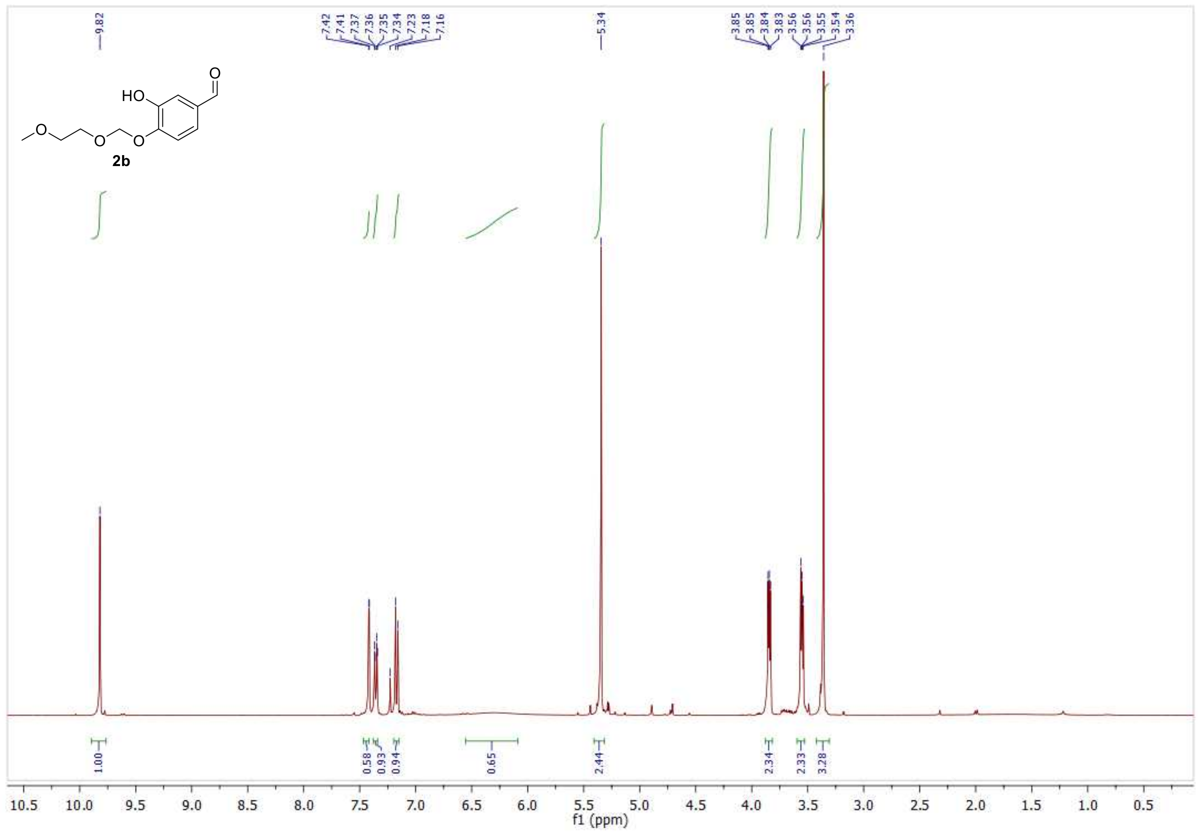

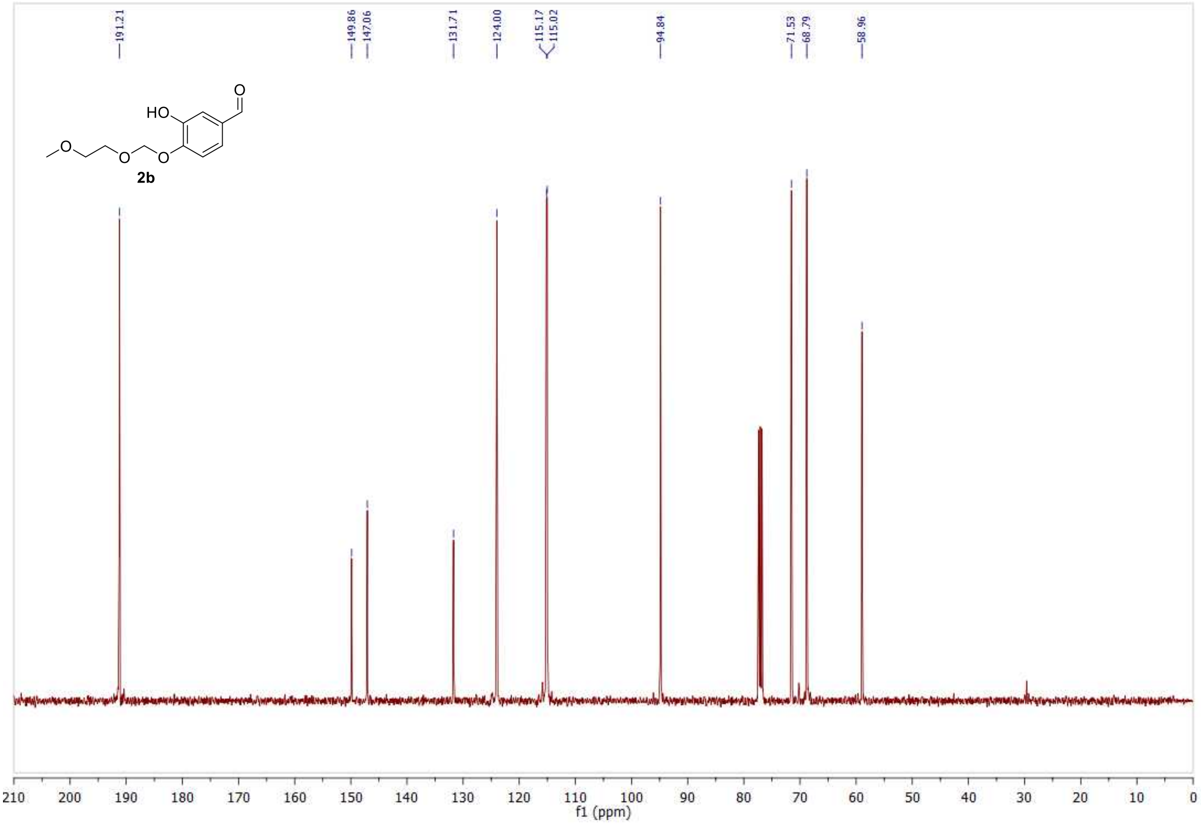

Figure S1: ^{1}H and ^{13}C NMR compound **2b**. 400 MHz, $CDCl_3$.

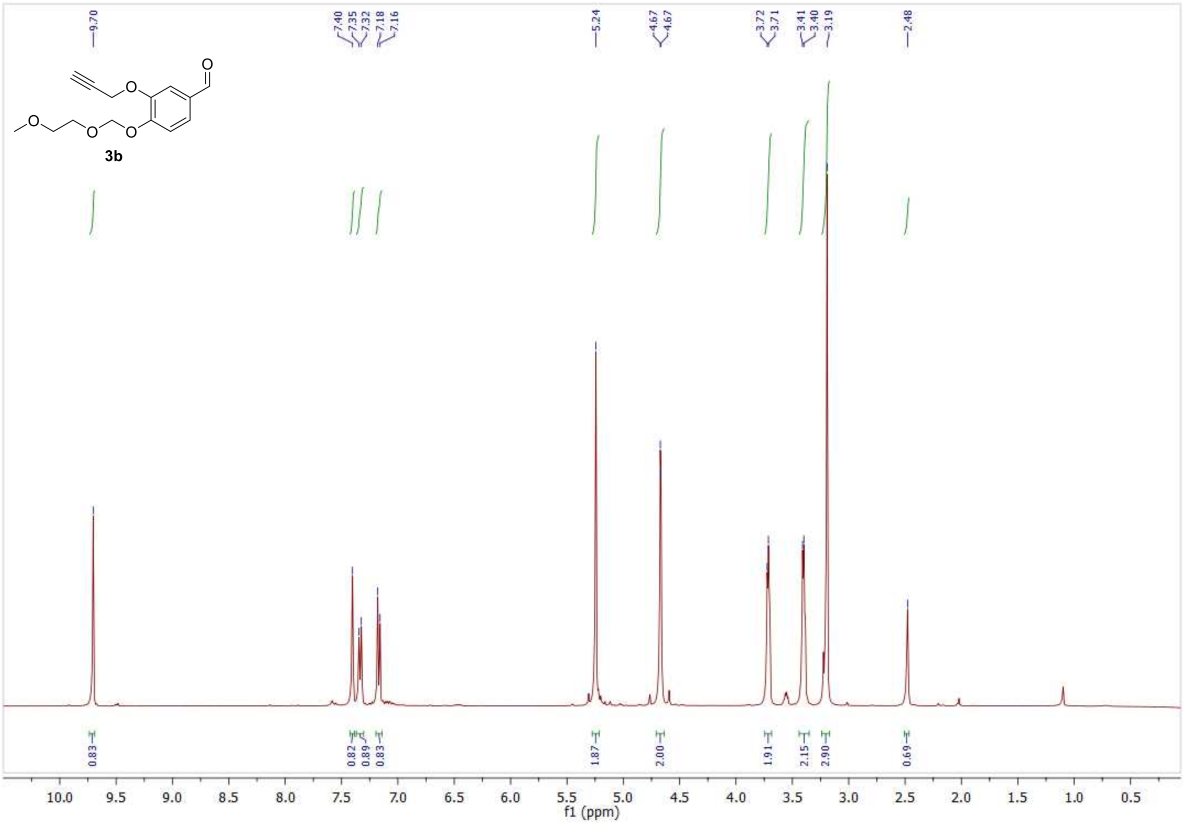

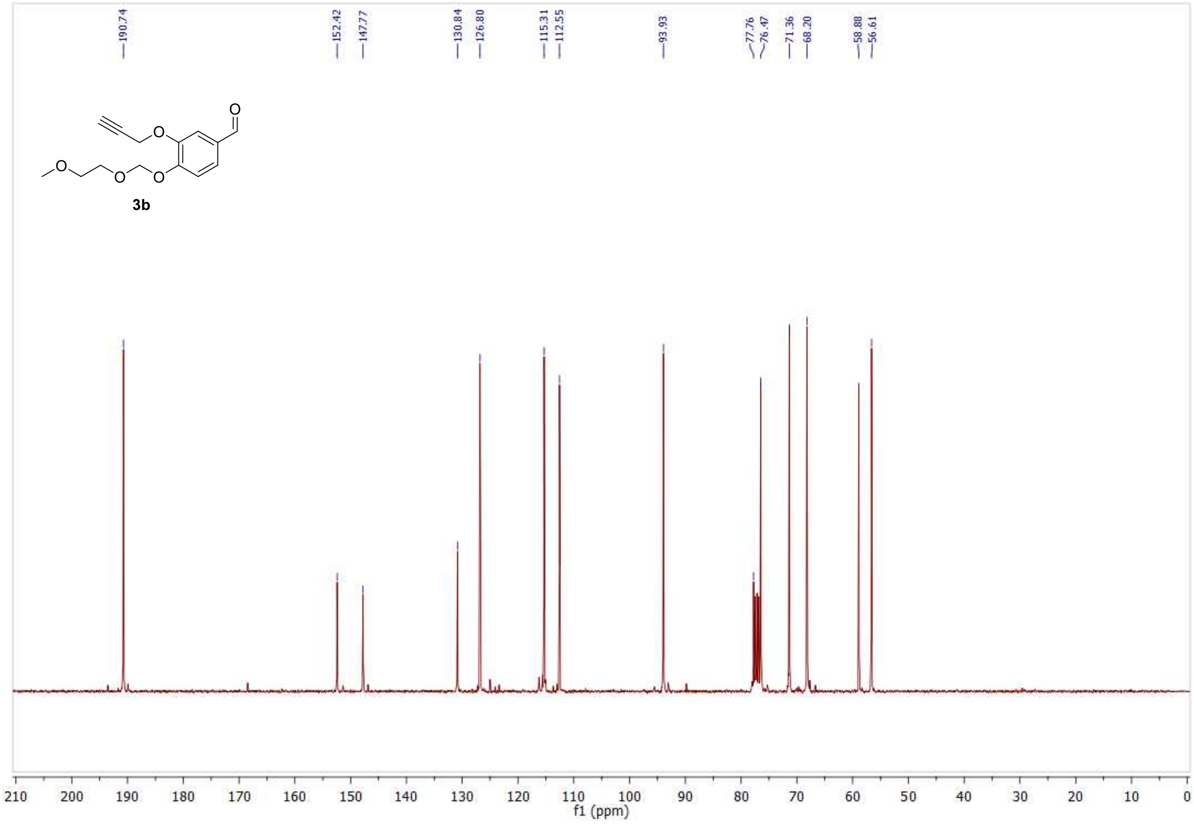

Figure S2: ^{1}H and ^{13}C NMR compound **3b**. 400 MHz, $CDCl_3$.

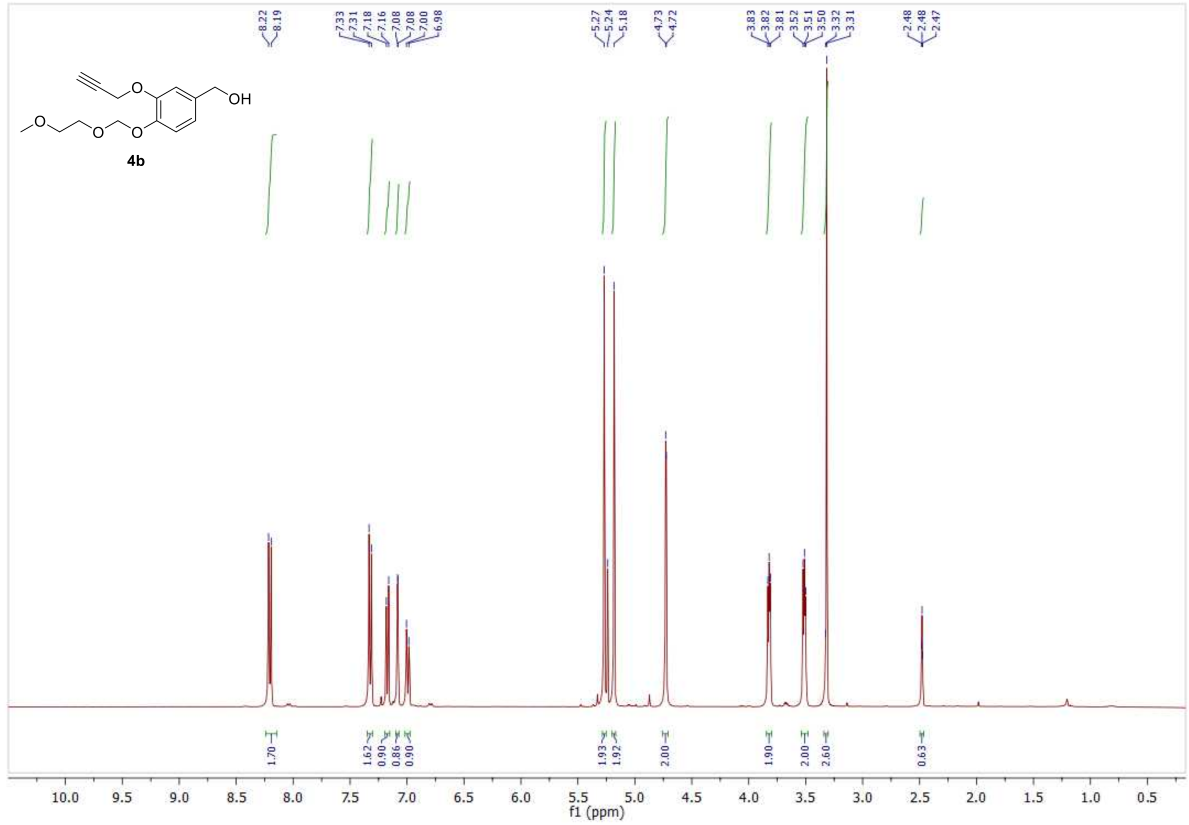

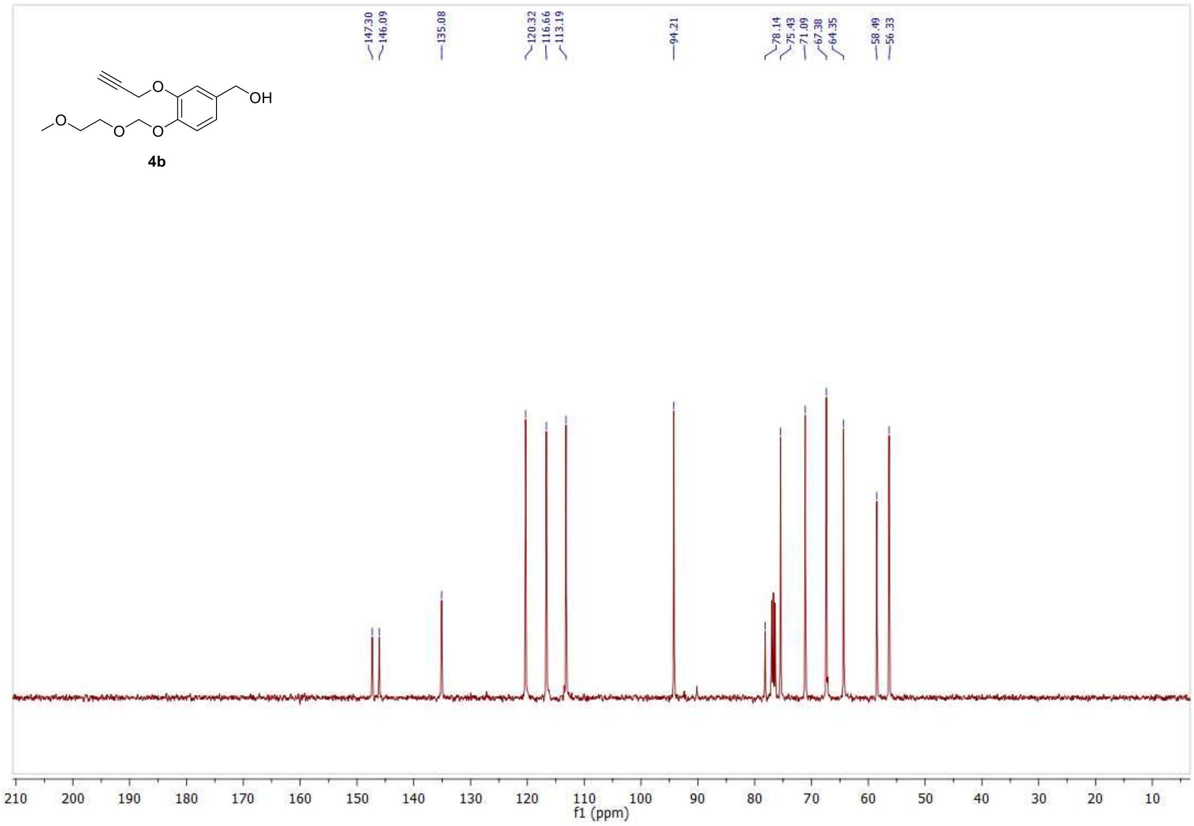

Figure S3: ^{1}H and ^{13}C NMR compound **4b**. 400 MHz, $CDCl_3$.

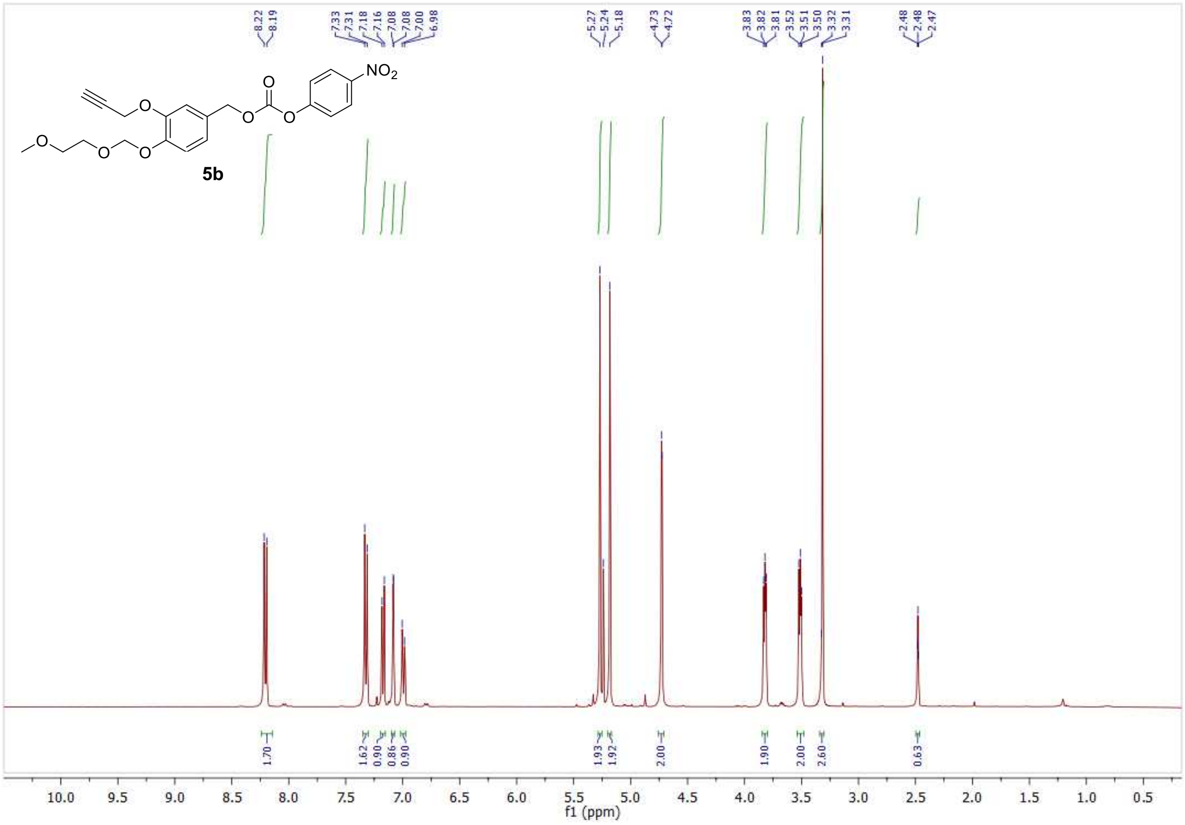

Figure S4: ^{1}H and compound **5b**. 400 MHz, $CDCl_3$.

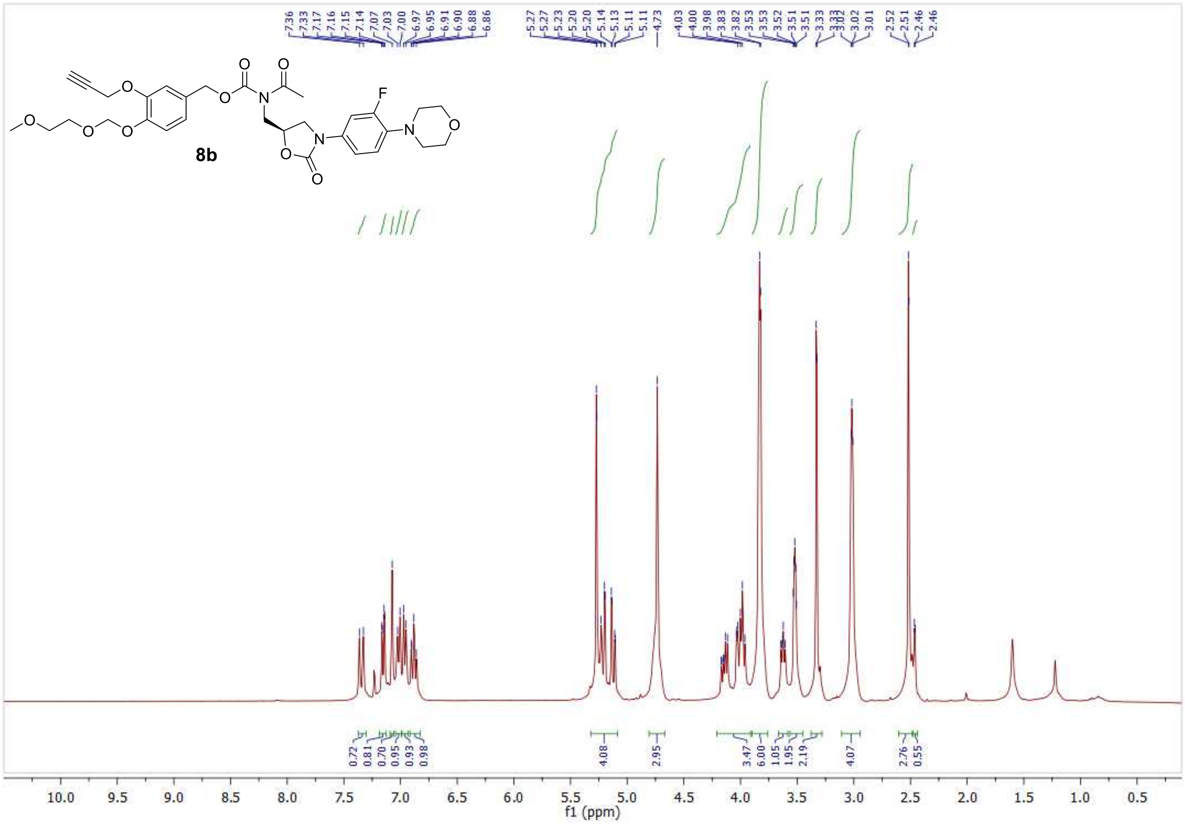

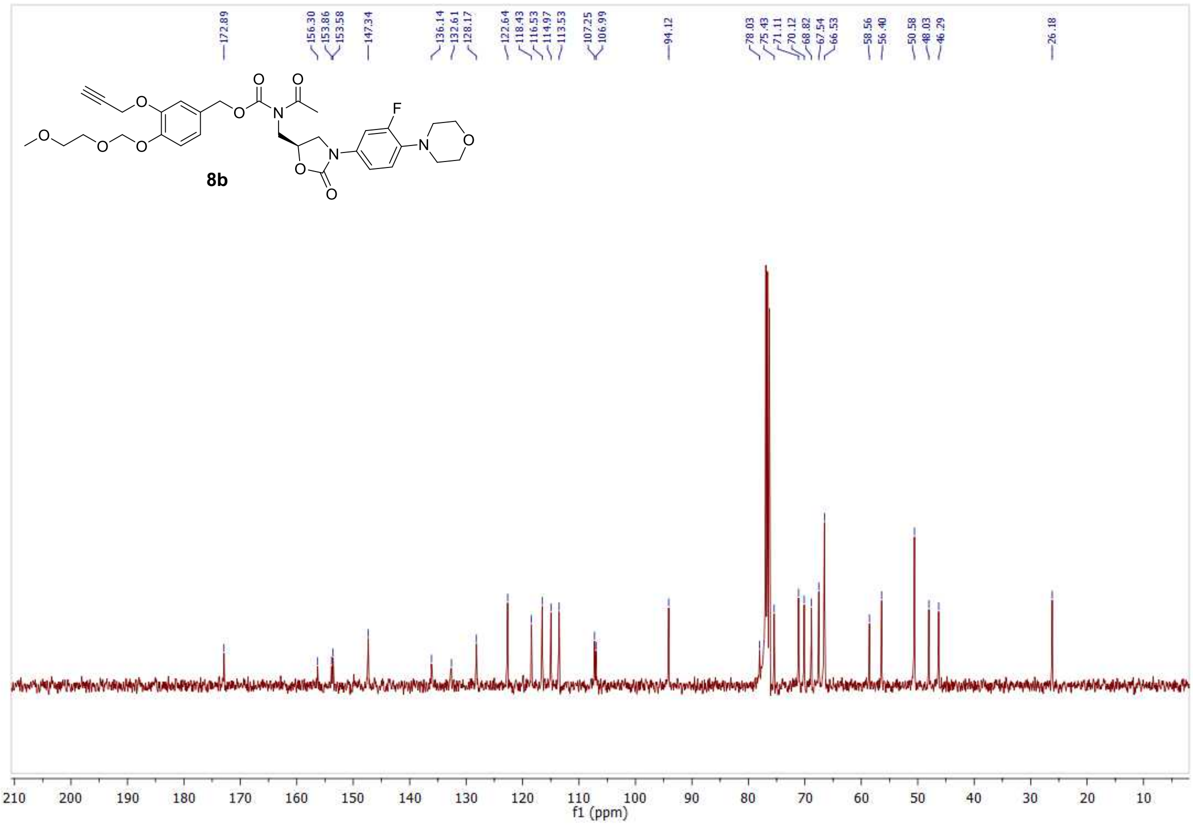

Figure S5: ^{1}H and ^{13}C NMR compound **8b**. 400 MHz, $CDCl_3$.

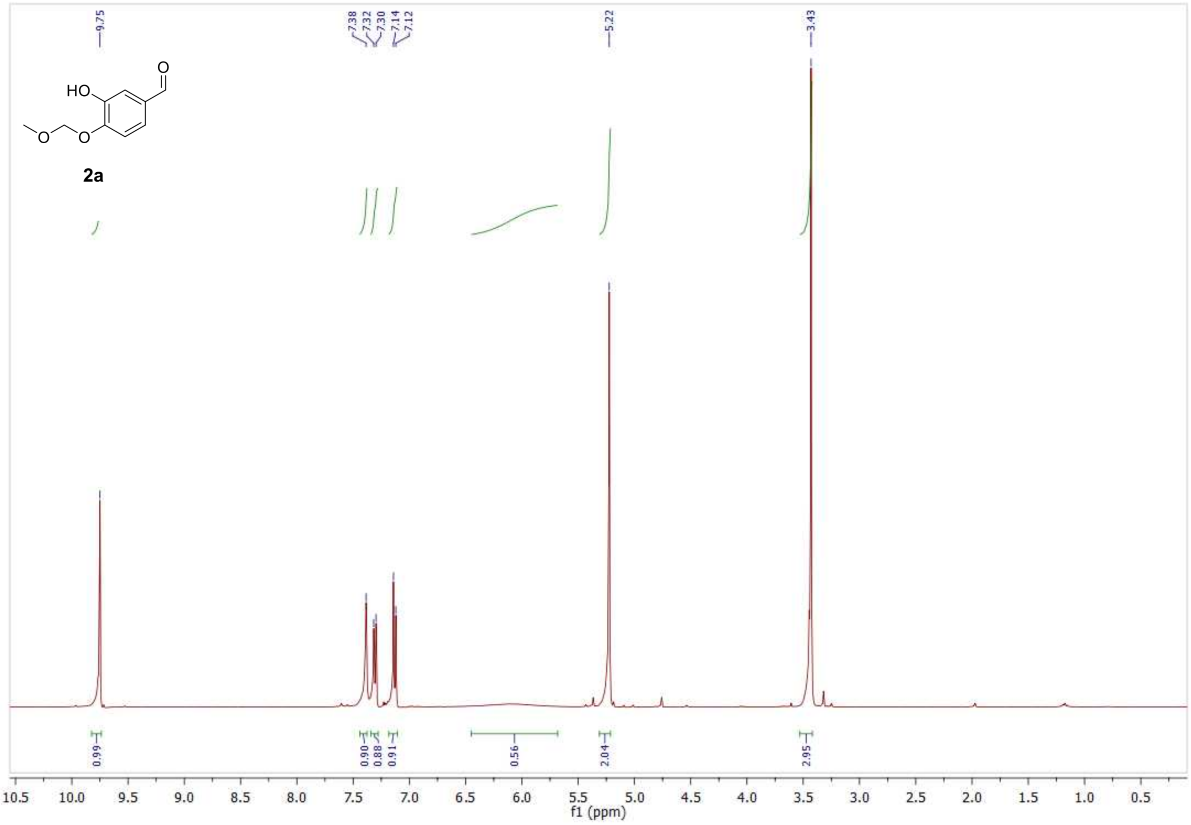

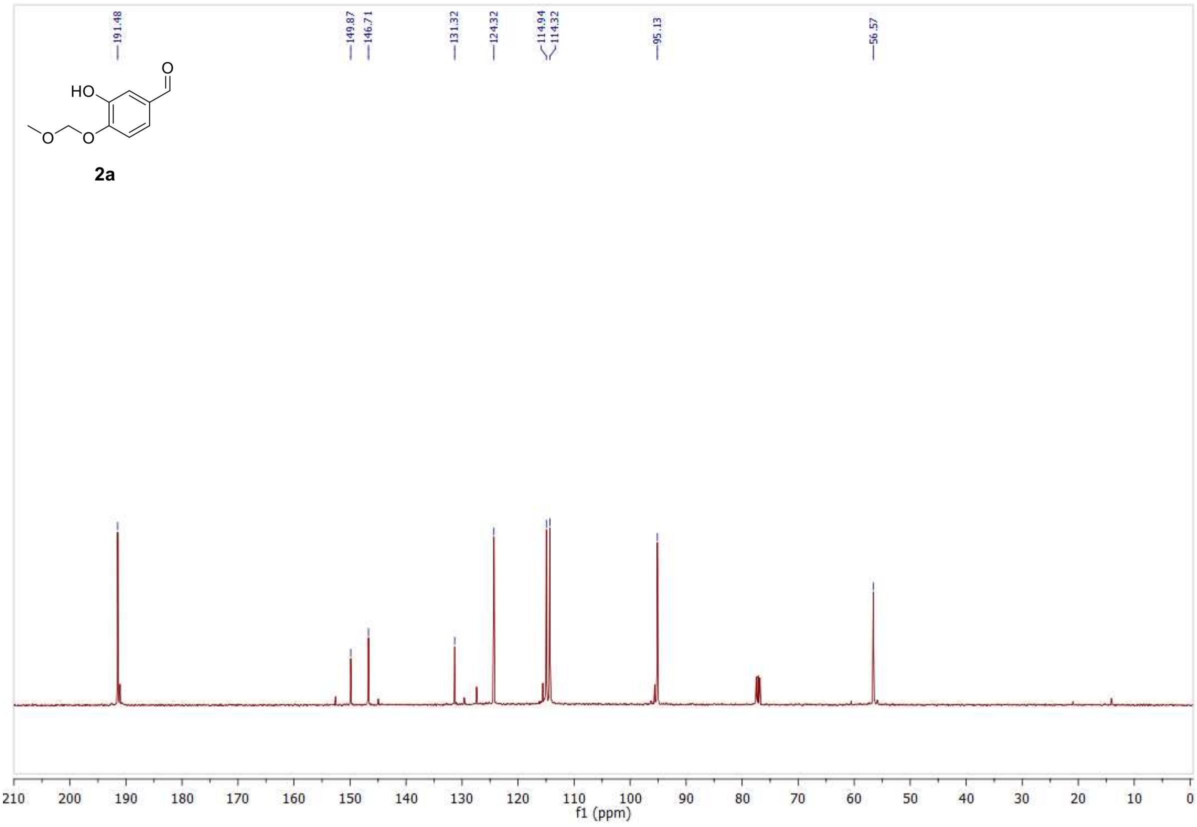

Figure S6: ^{1}H and ^{13}C NMR compound **2a** 400 MHz, $CDCl_3$.

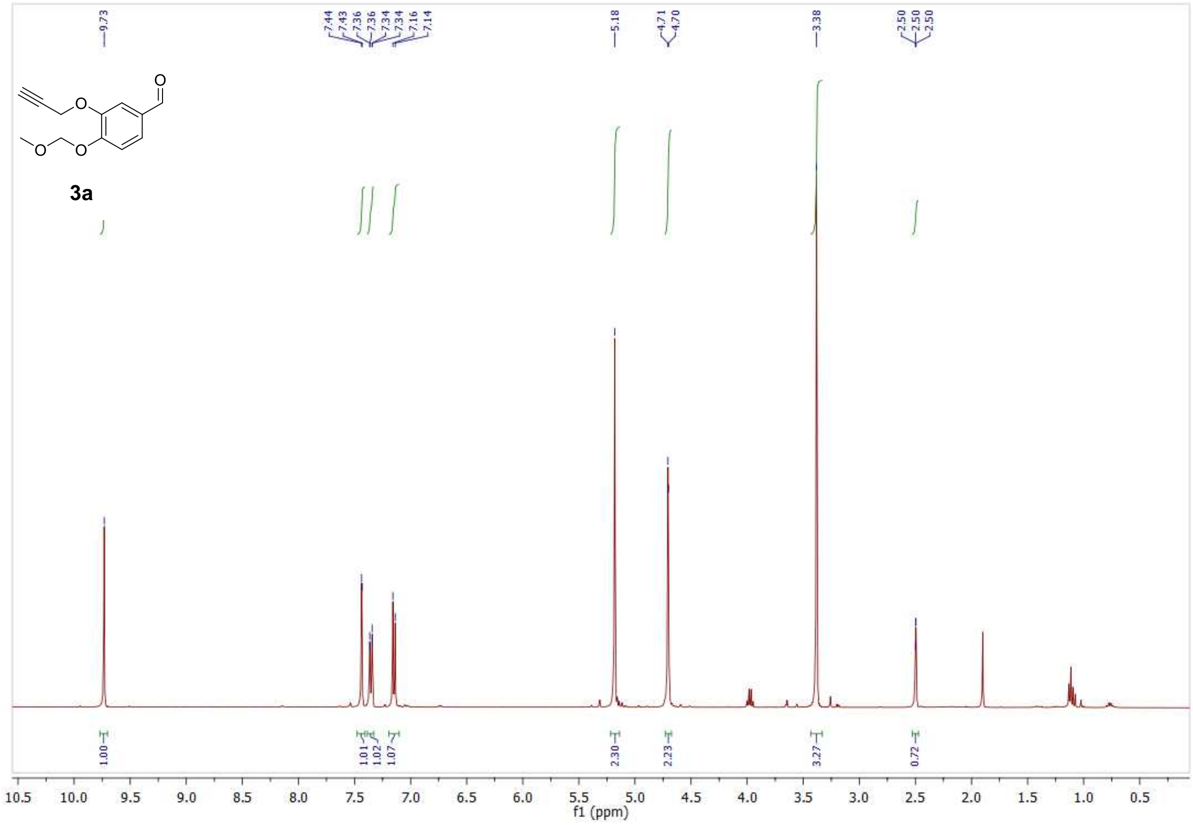

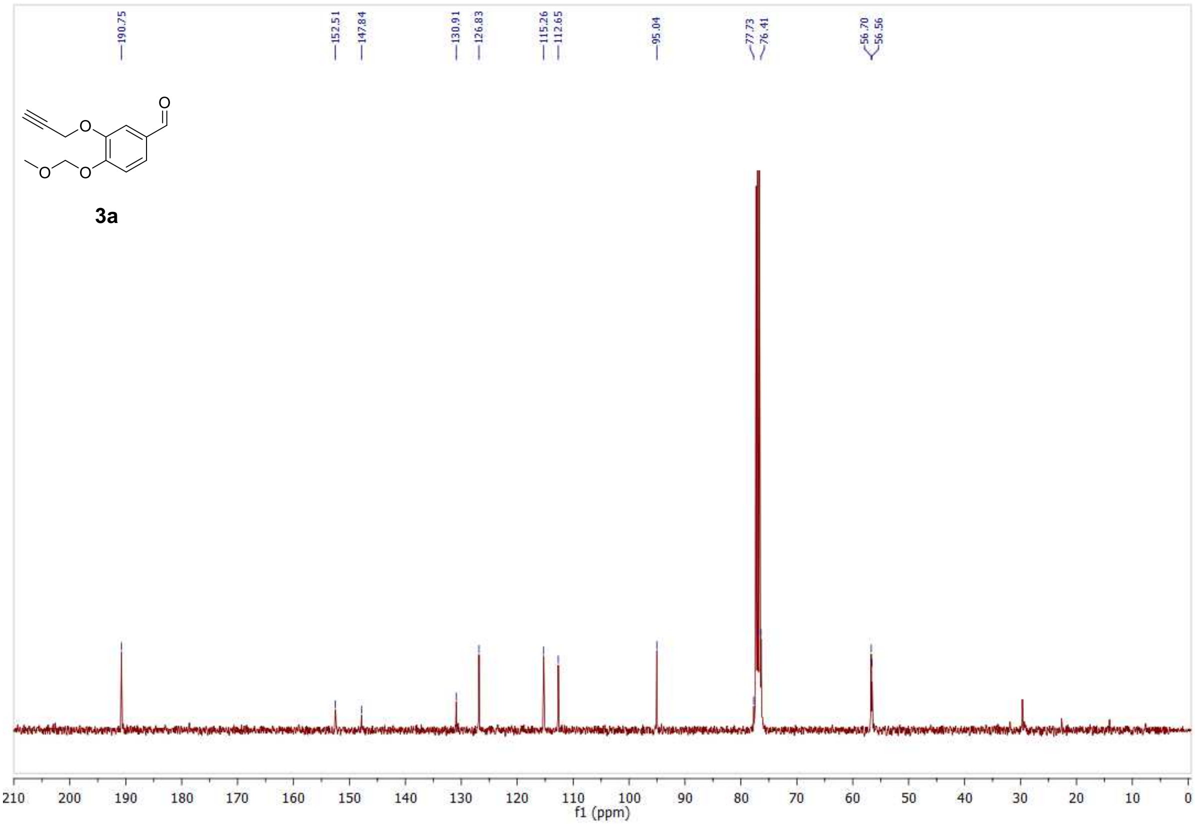

Figure S7: ^{1}H and ^{13}C NMR compound **3a**. 400 MHz, $CDCl_3$.

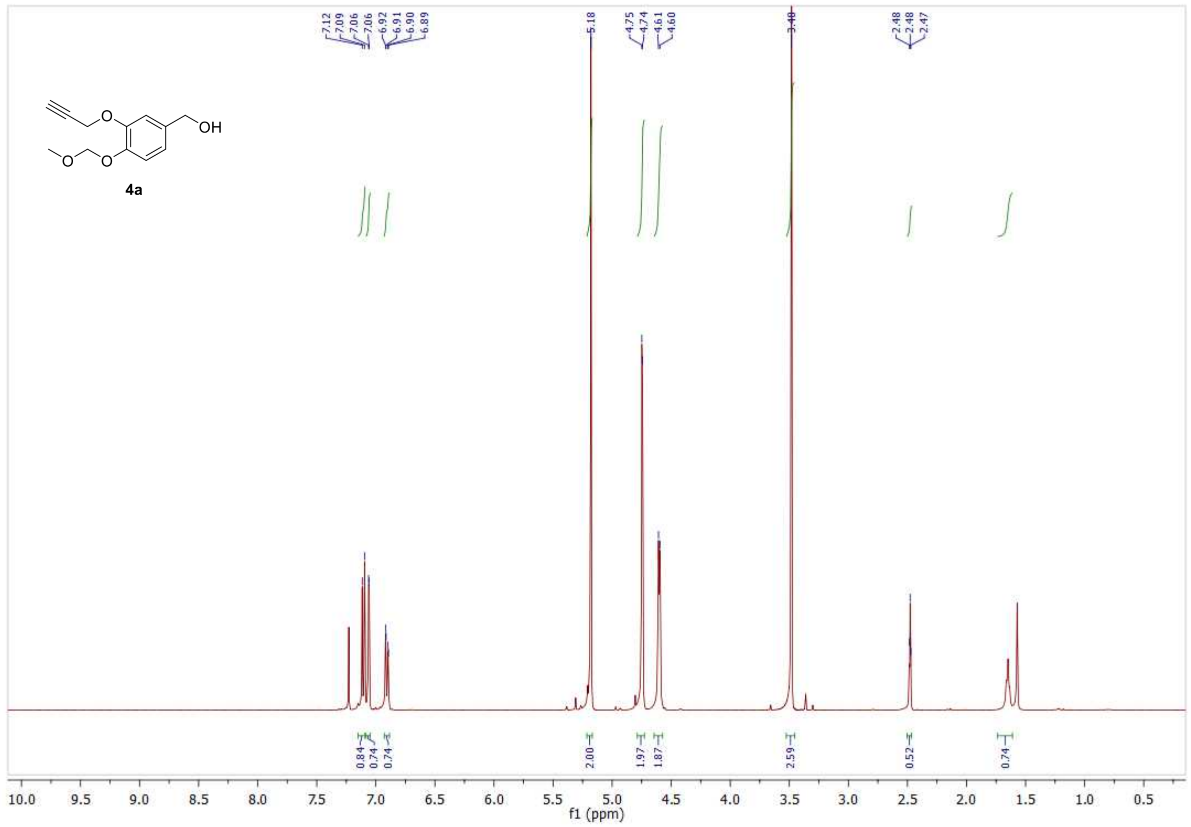

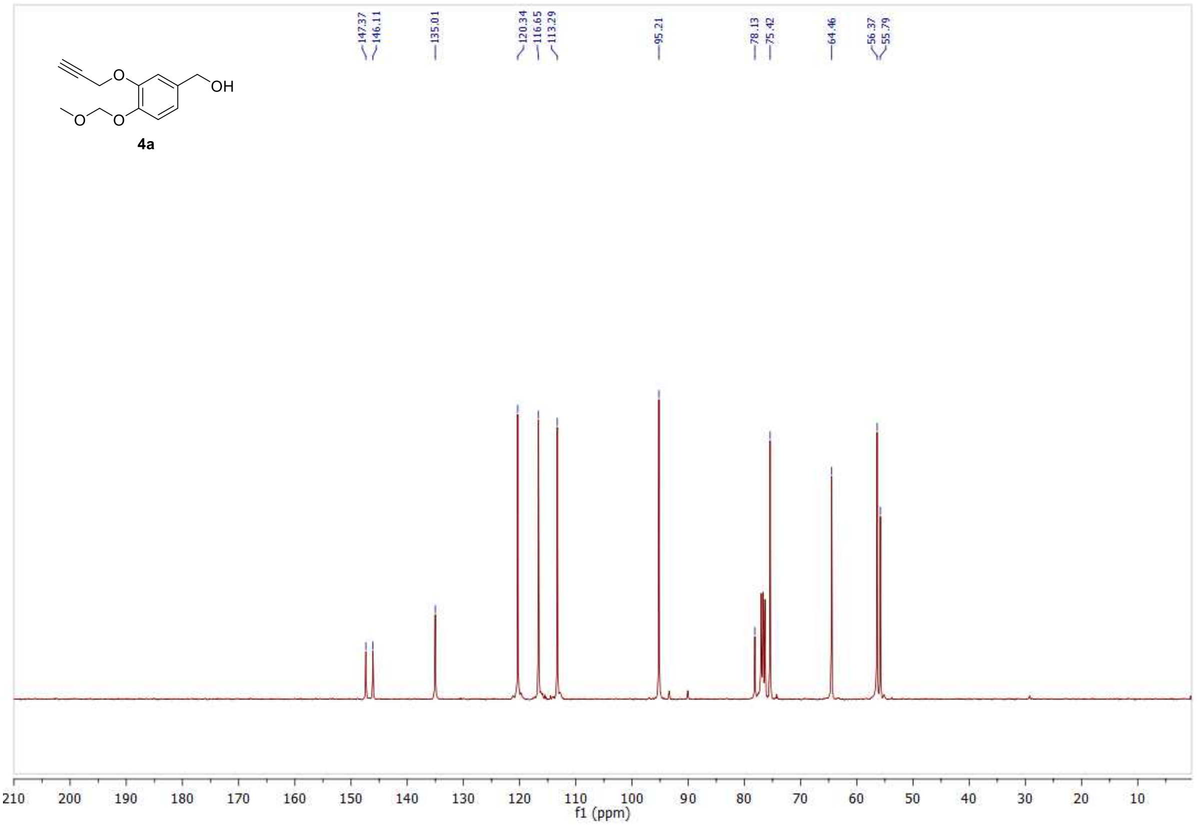

Figure S8: ^{1}H and ^{13}C NMR compound **4a**. 400 MHz, $CDCl_3$.

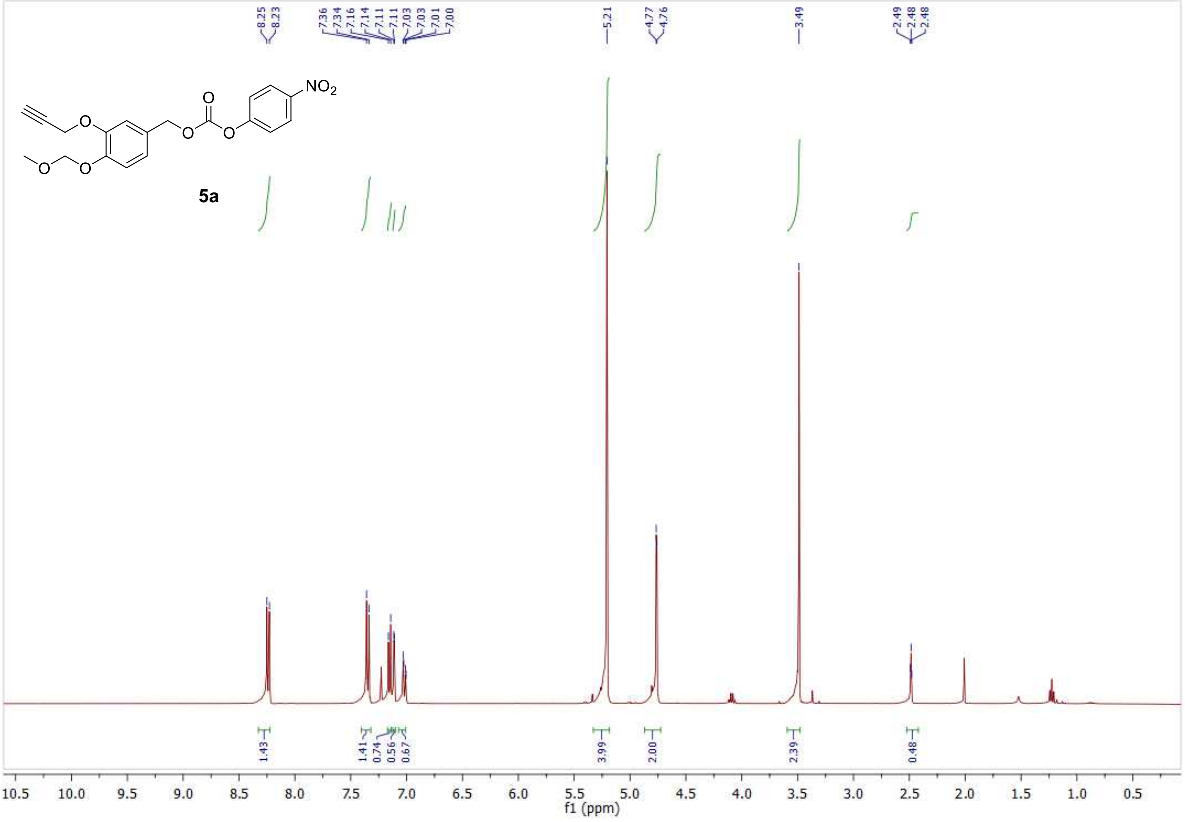

Figure S9: ^{1}H NMR compound **5a**. 400 MHz, $CDCl_3$.

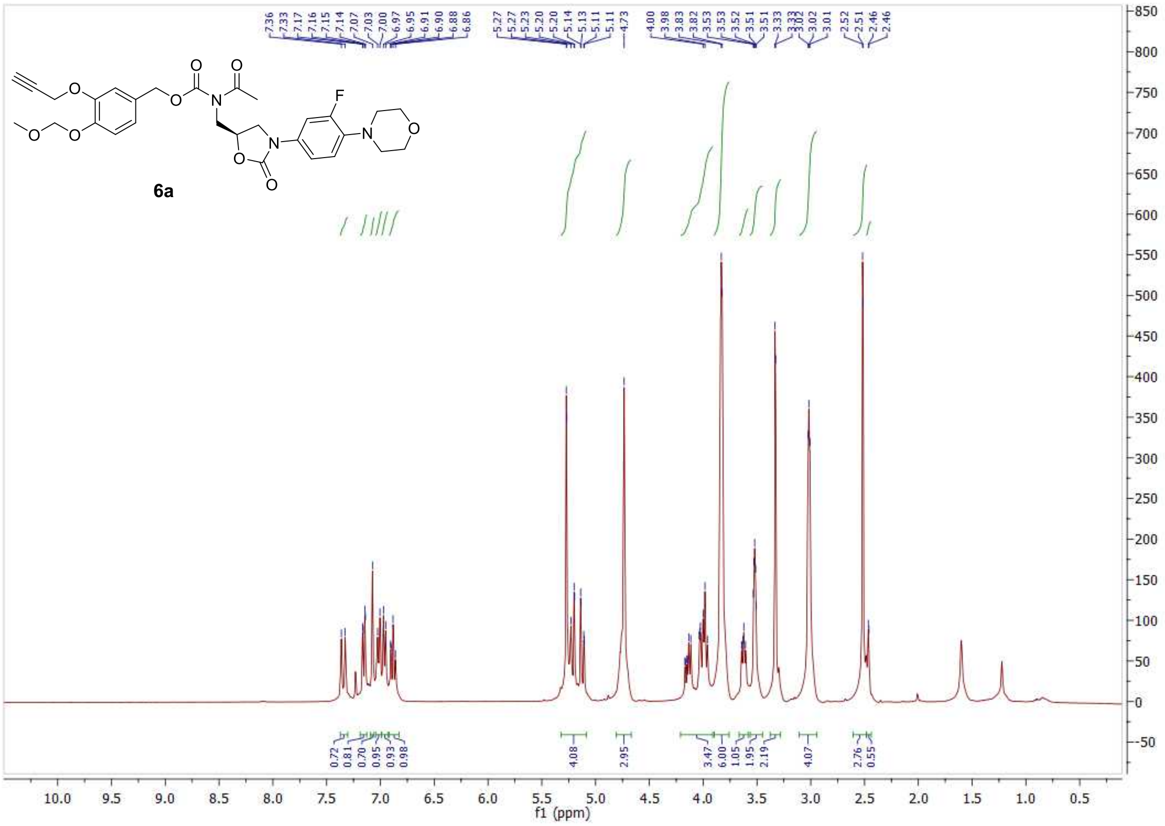

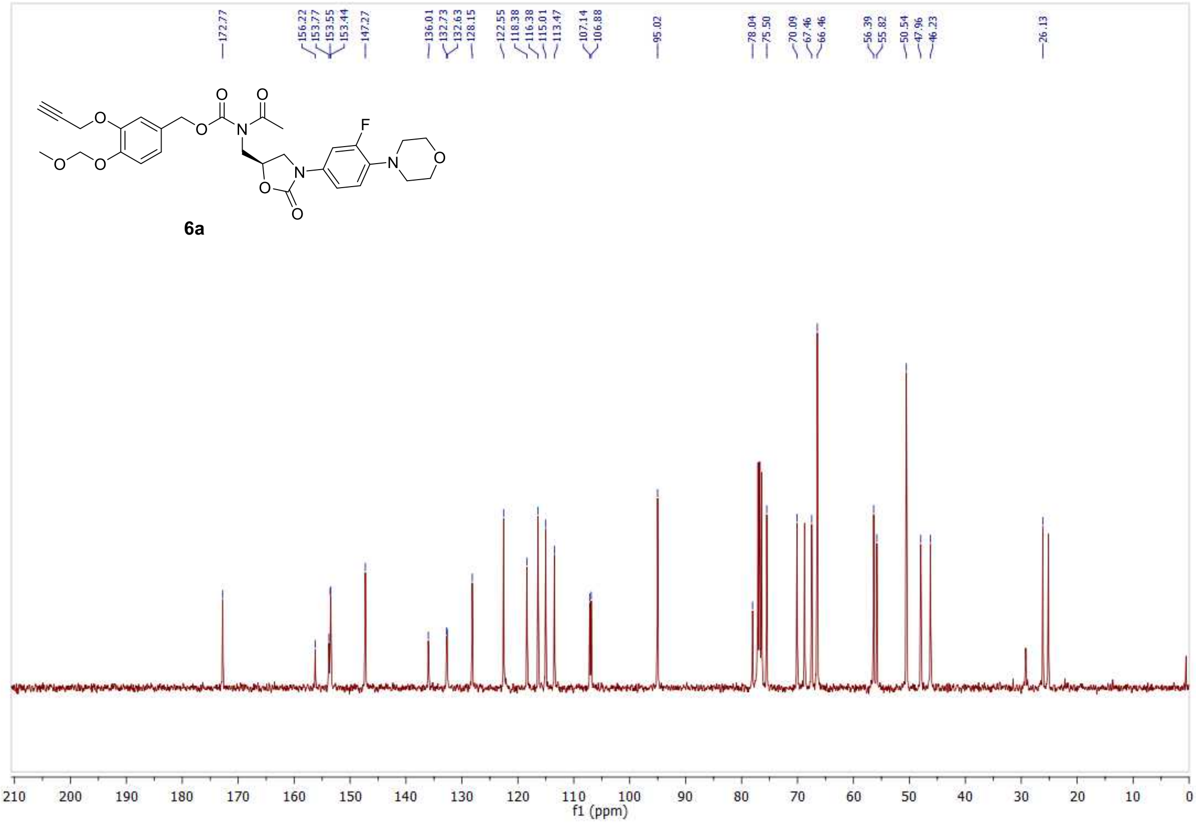

Figure S10: ^{1}H and ^{13}C NMR compound **6a**. 400 MHz, $CDCl_3$.

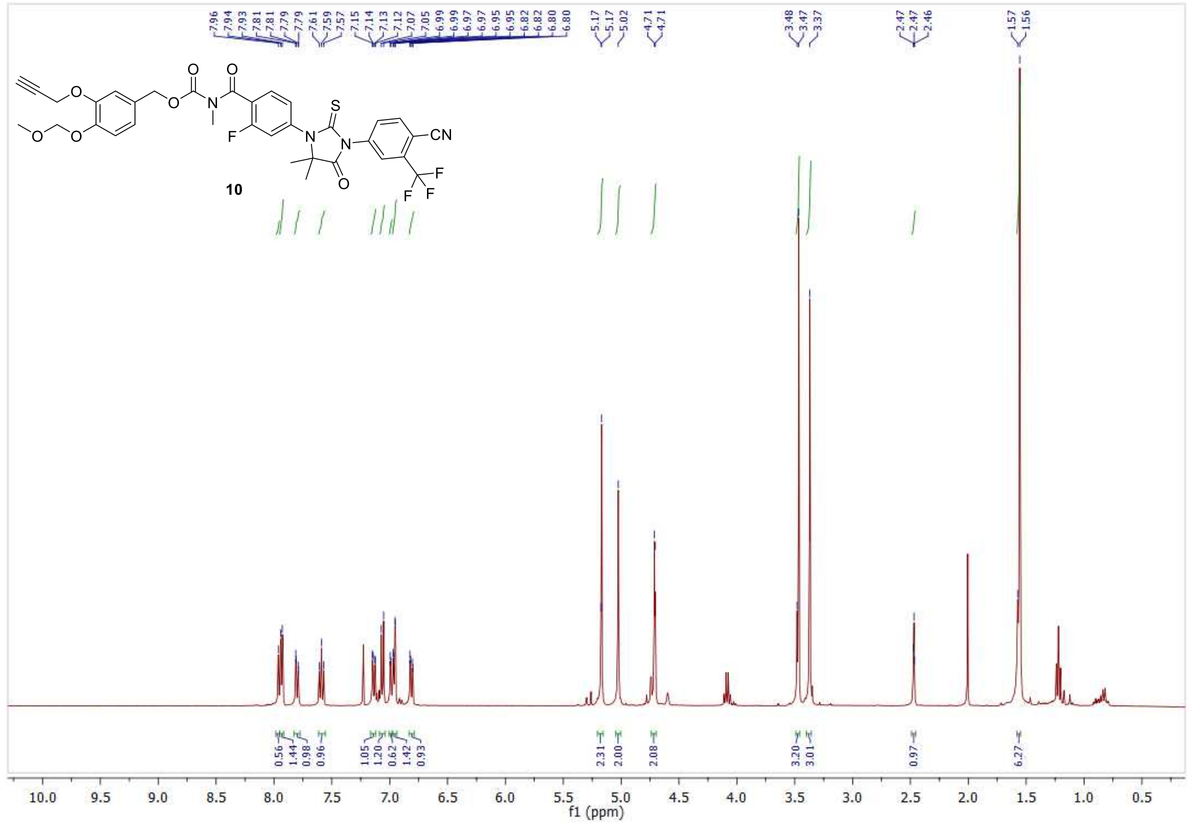

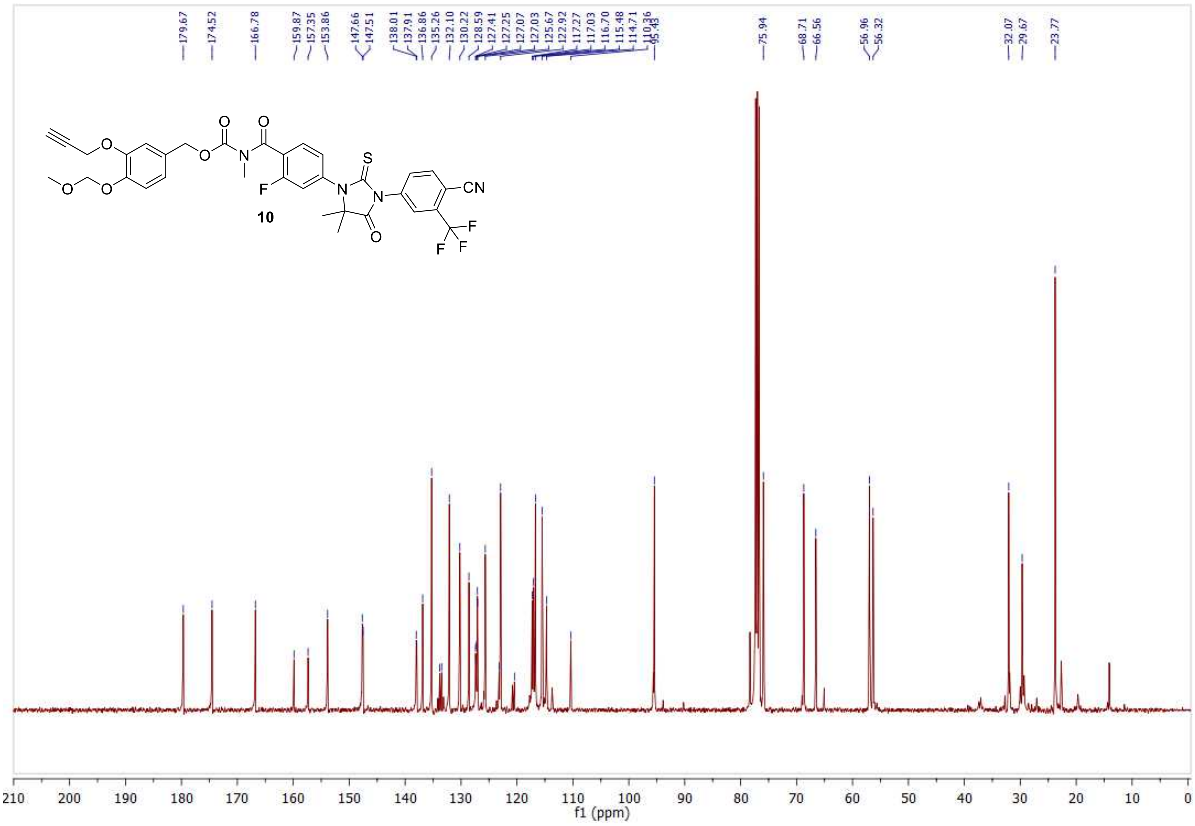

Figure S11: ^{1}H and ^{13}C NMR compound **10**. 400 MHz, $CDCl_3$.

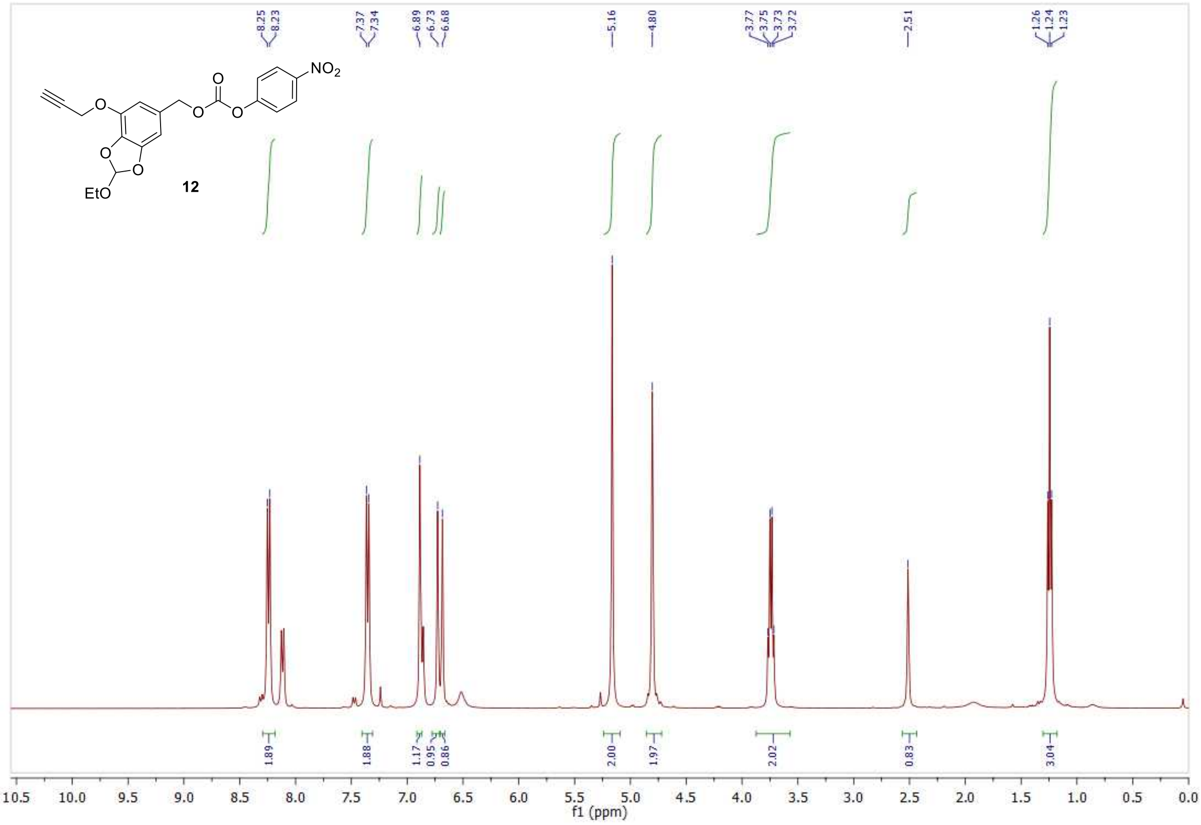

Figure S12: ^{1}H NMR compound **12**. 400 MHz, $CDCl_3$.

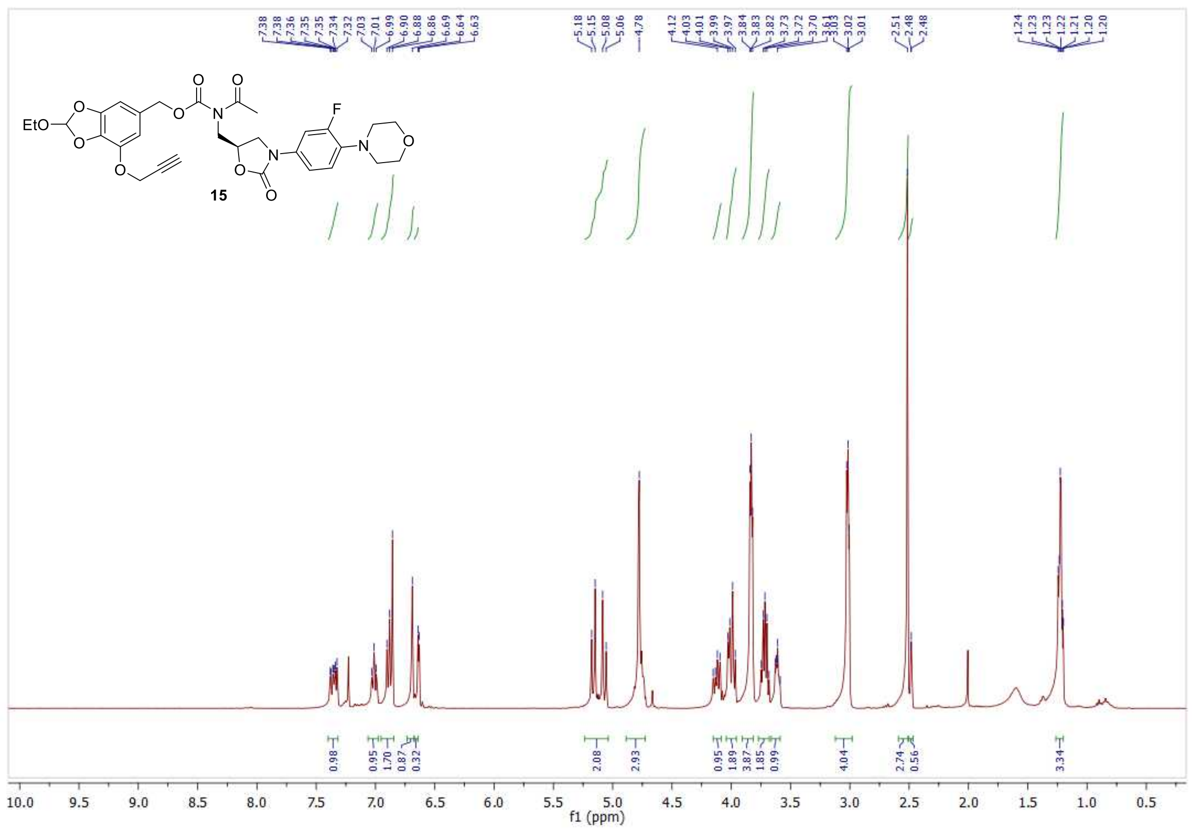

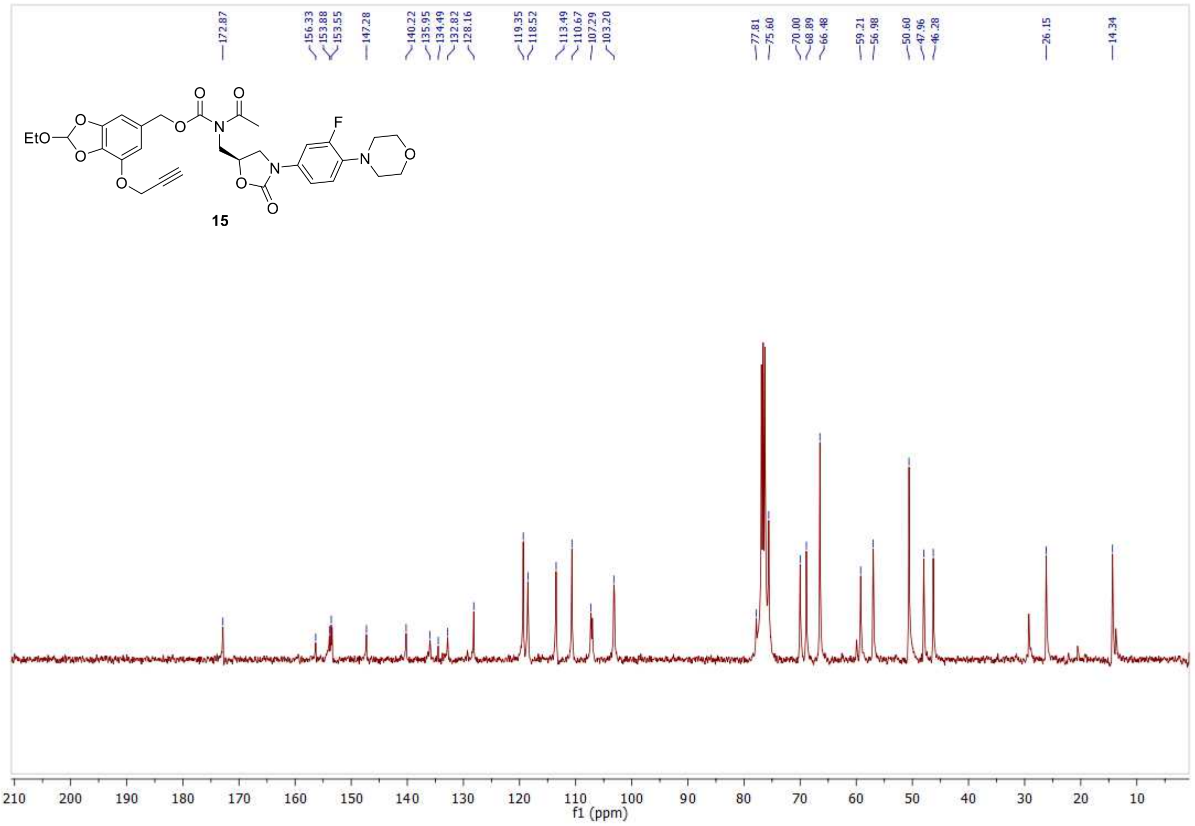

Figure S13: ^{1}H and ^{13}C NMR compound **15**. 400 MHz, $CDCl_3$.

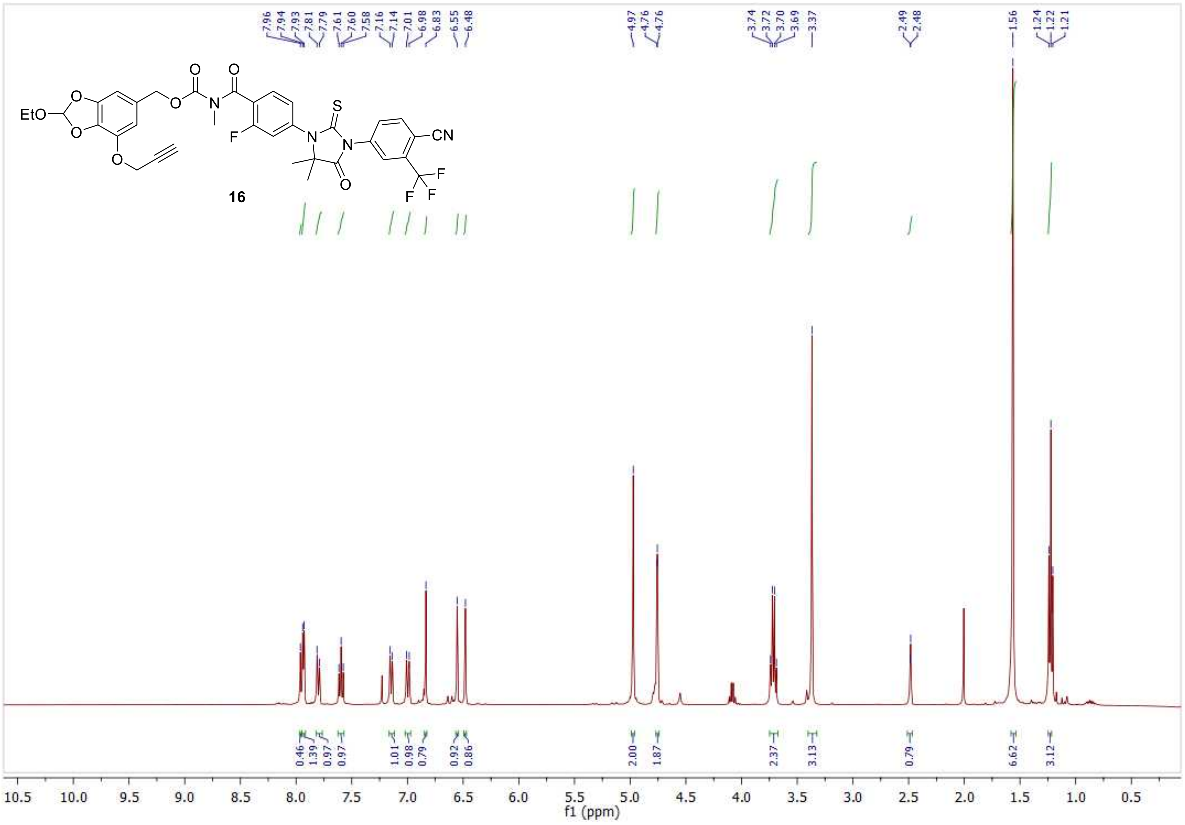

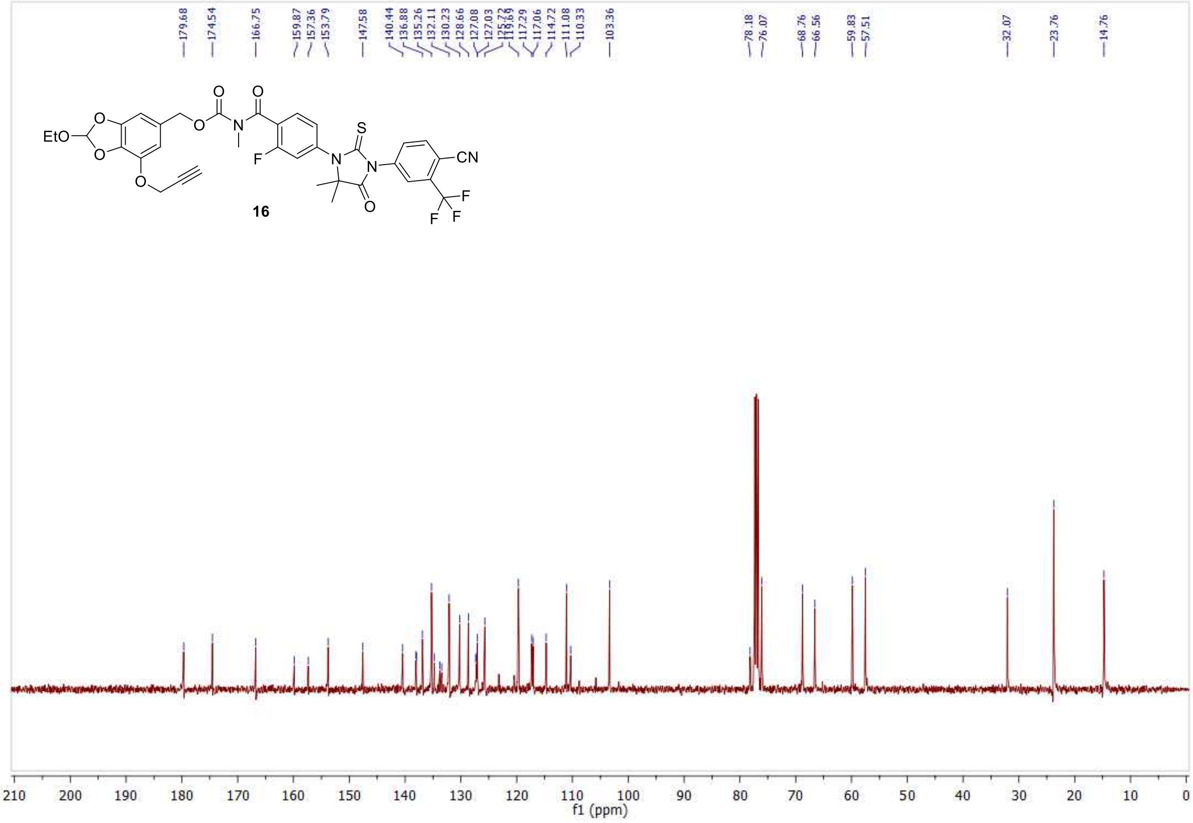

Figure S14: ^{1}H and ^{13}C NMR compound **16**. 400 MHz, $CDCl_3$.

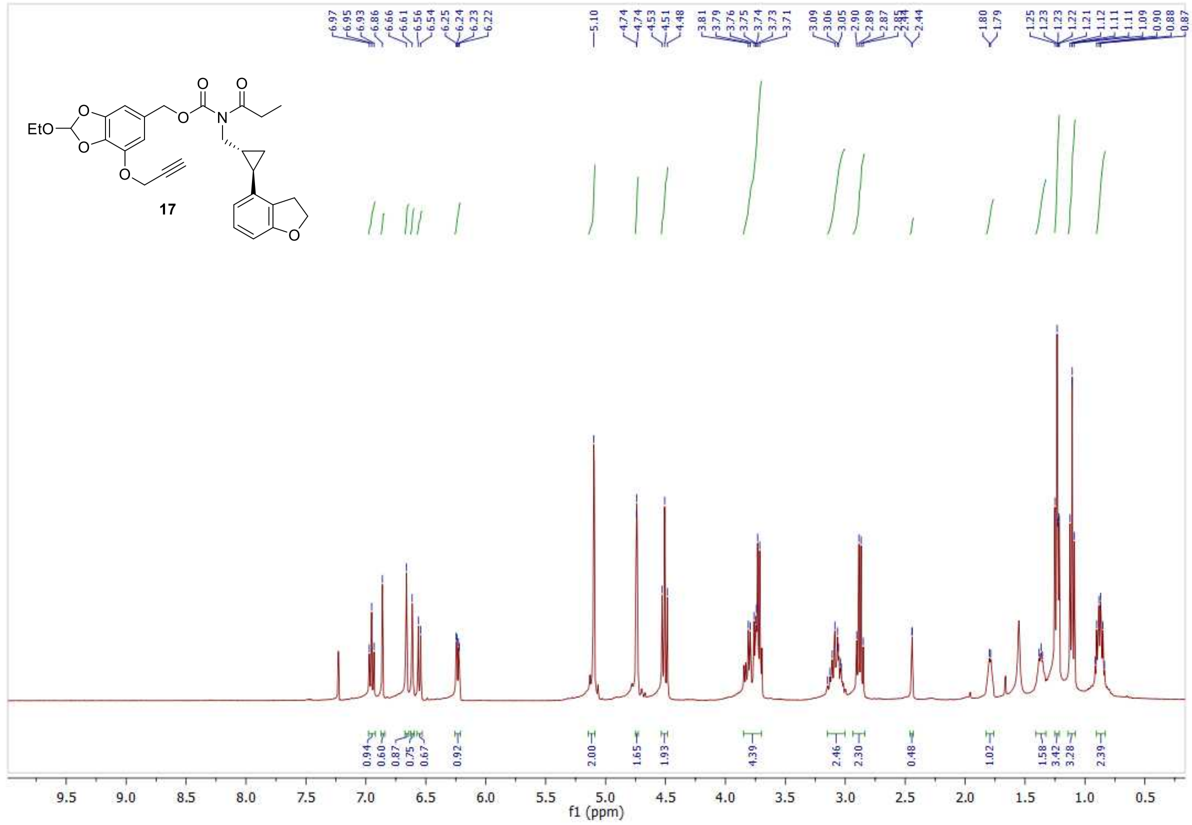

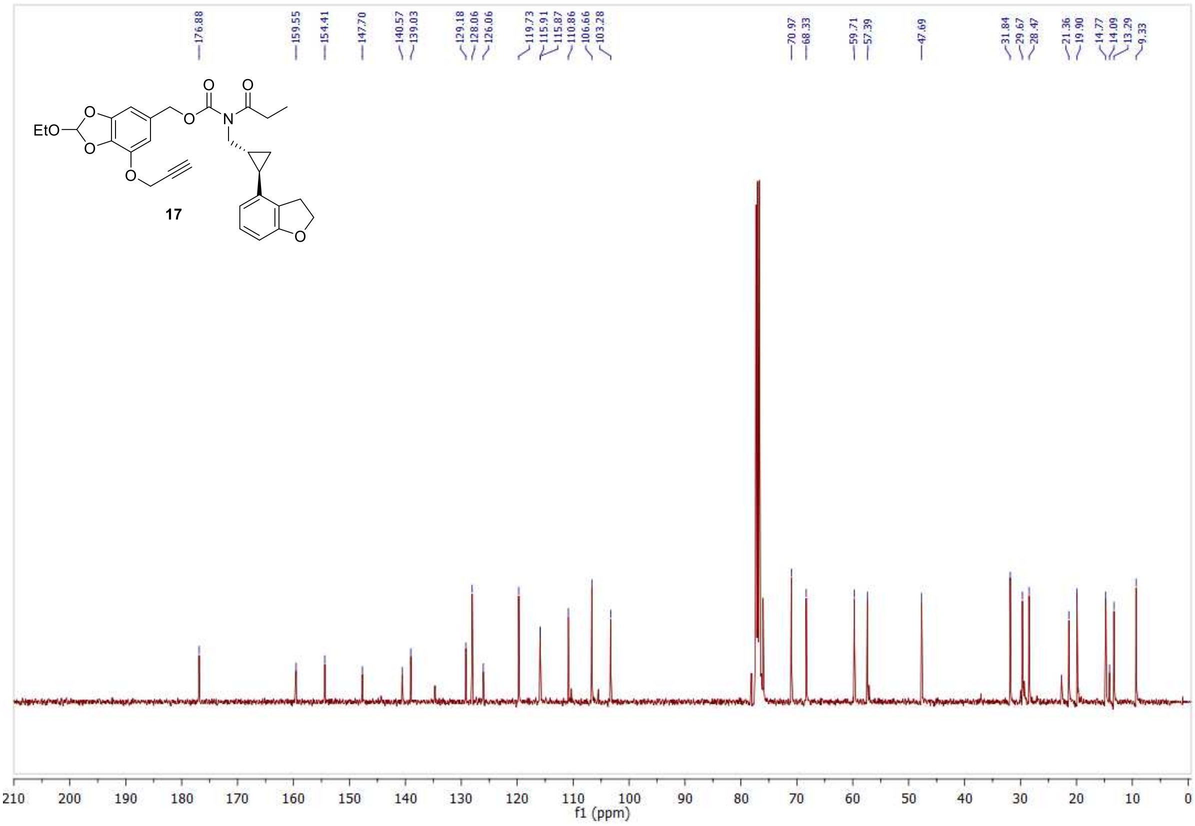

Figure S15: ^{1}H and ^{13}C NMR compound **17**. 400 MHz, $CDCl_3$.

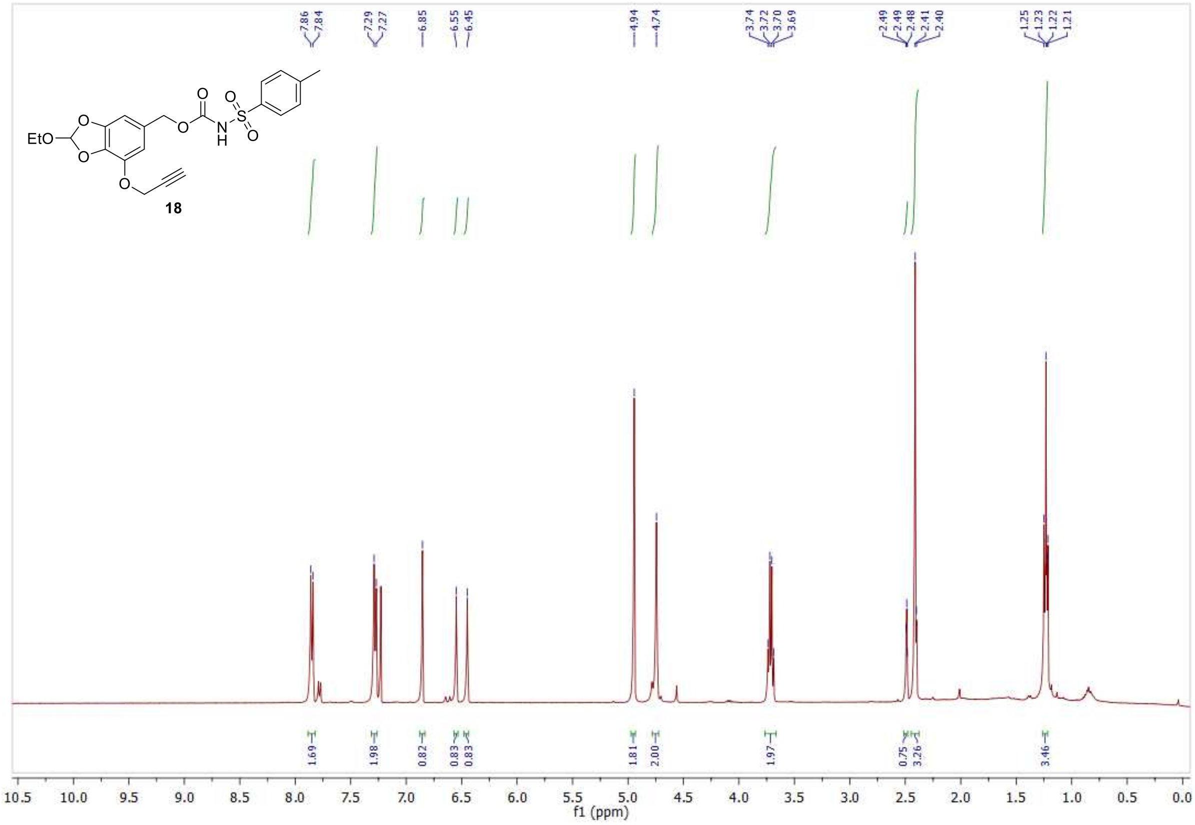

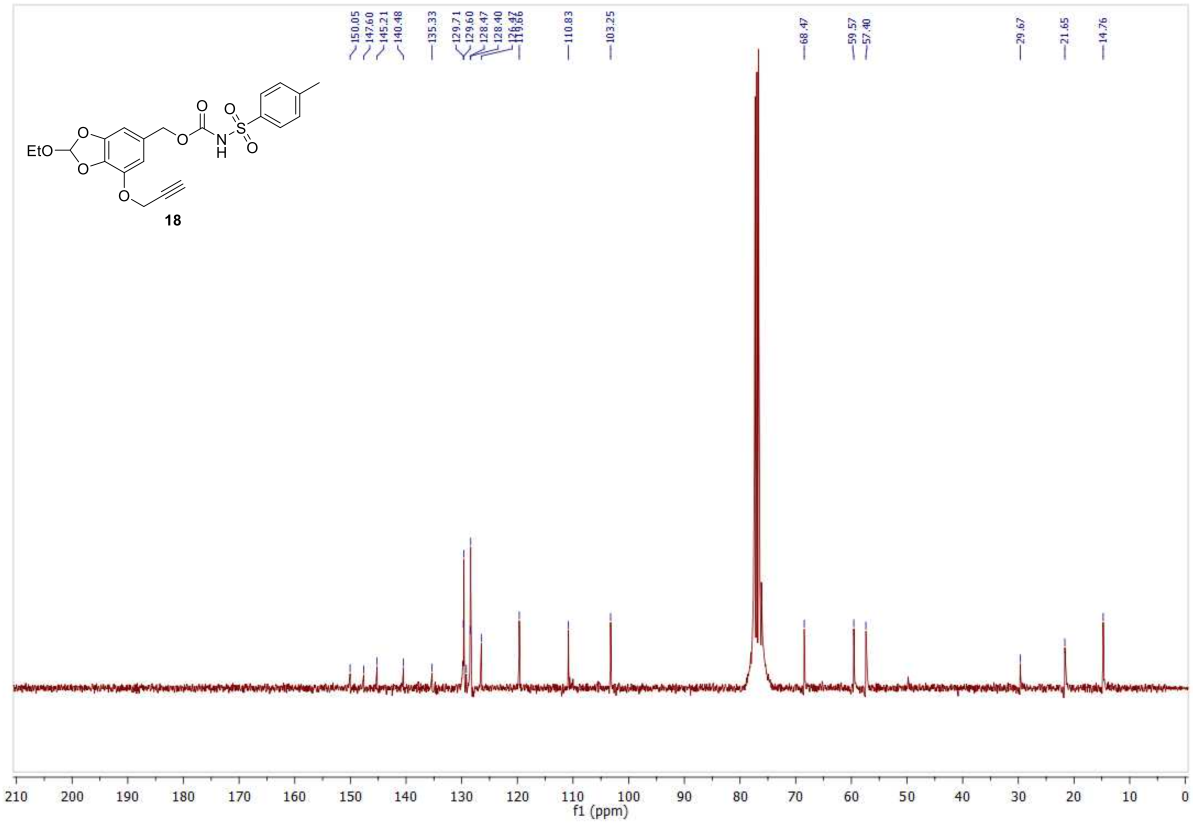

Figure S16: ^{1}H and ^{13}C NMR compound **18**. 400 MHz, $CDCl_3$.

HPLC-MS Spectra (S17-S25)

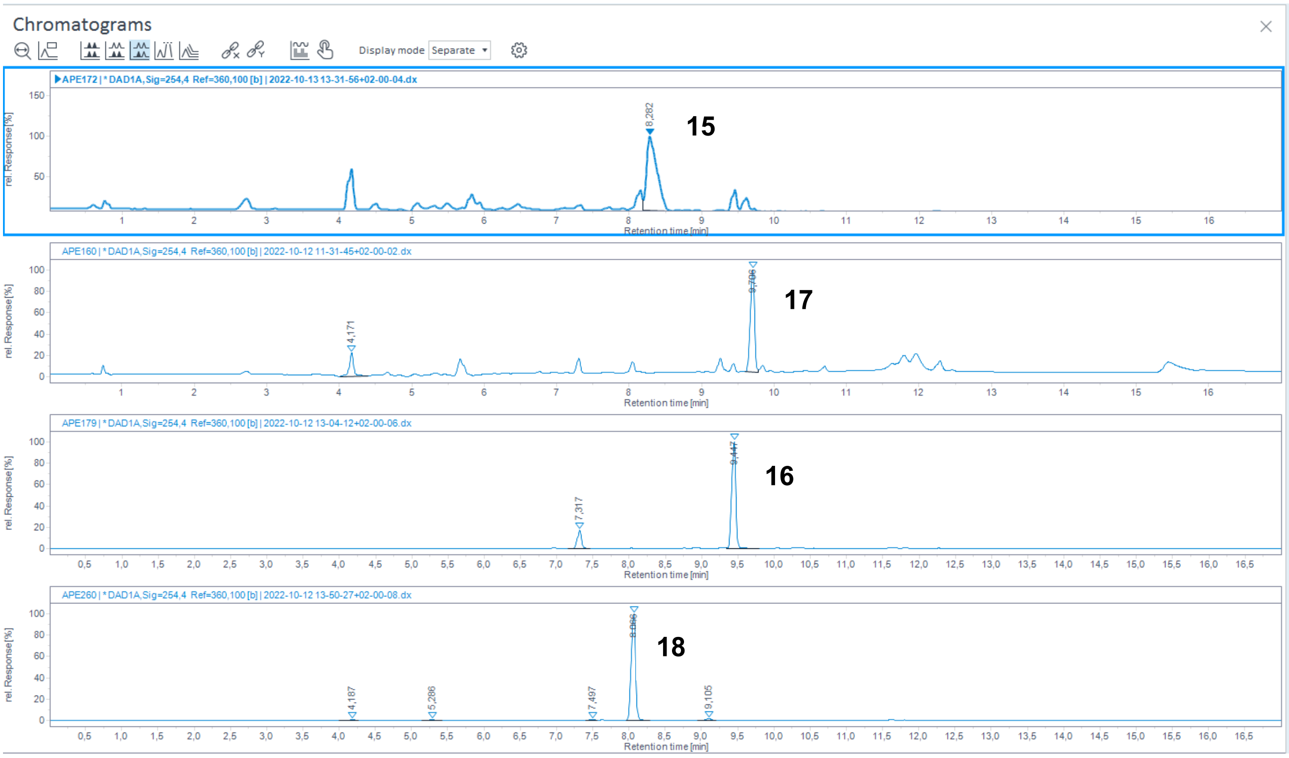

Figure S17. HPLC-MS of pure compounds **15-18**.

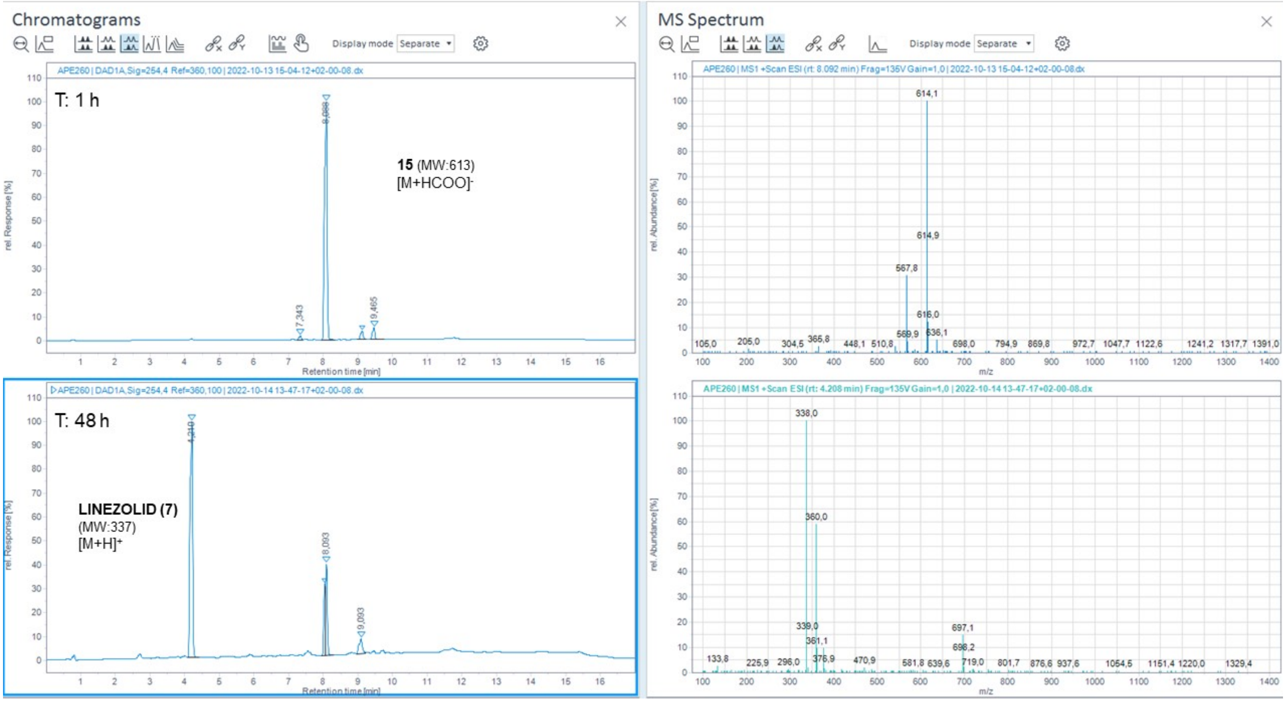

Figure S18: Hydrolysis of compound **15** at pH 5.5. Time 1 h and 48 h

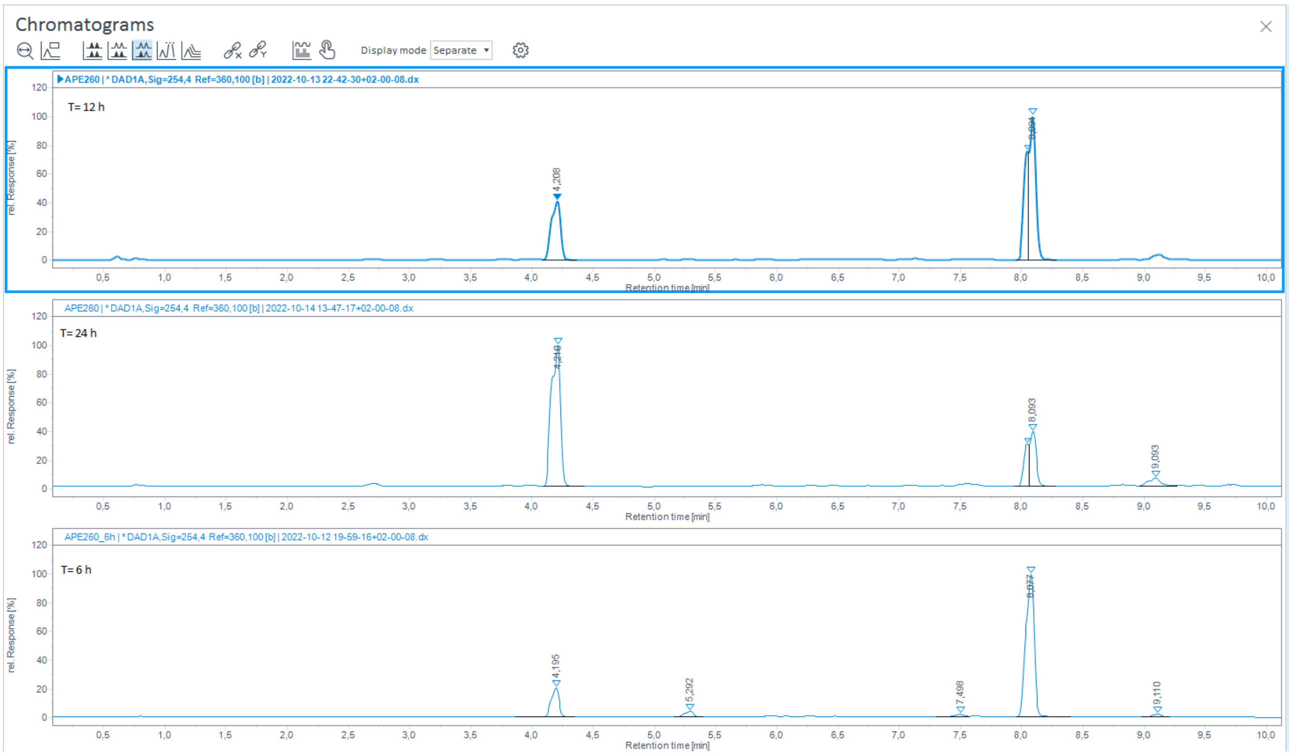

Figure S19: Hydrolysis of compound **15** at different times (6 h,12 h and 24 h)

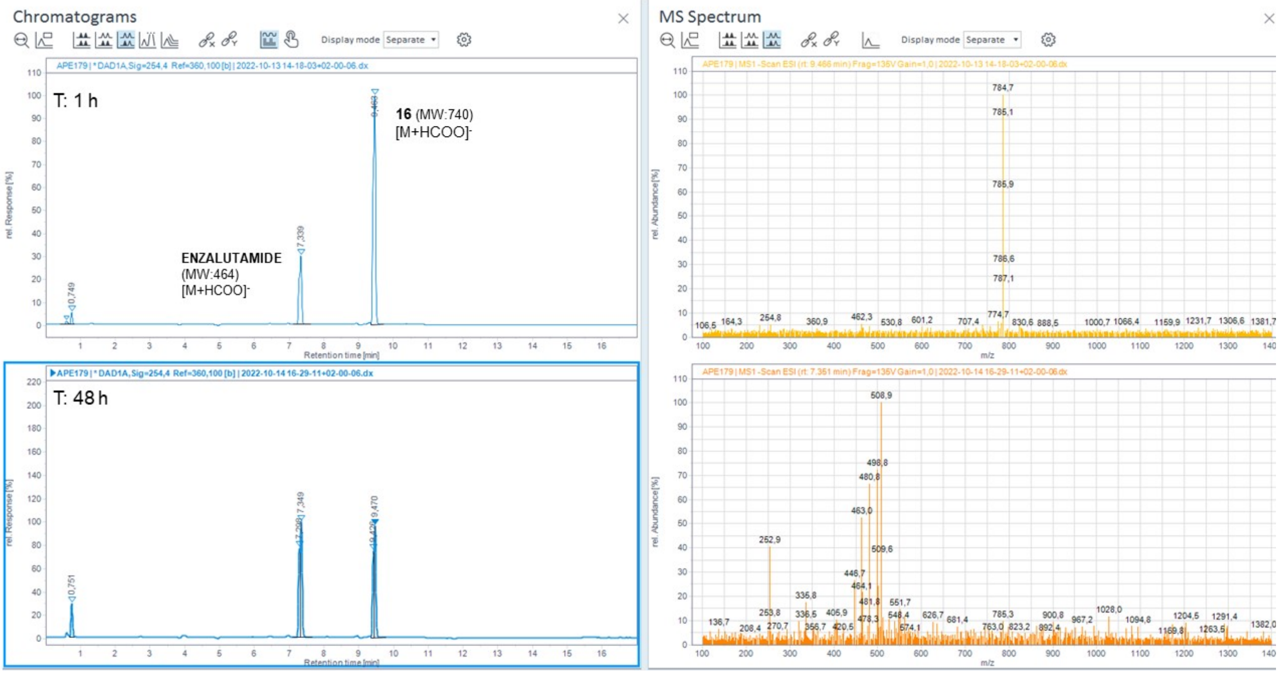

Figure S20: Hydrolysis of compound **16** at pH 5.5. Time 1 h and 48 h

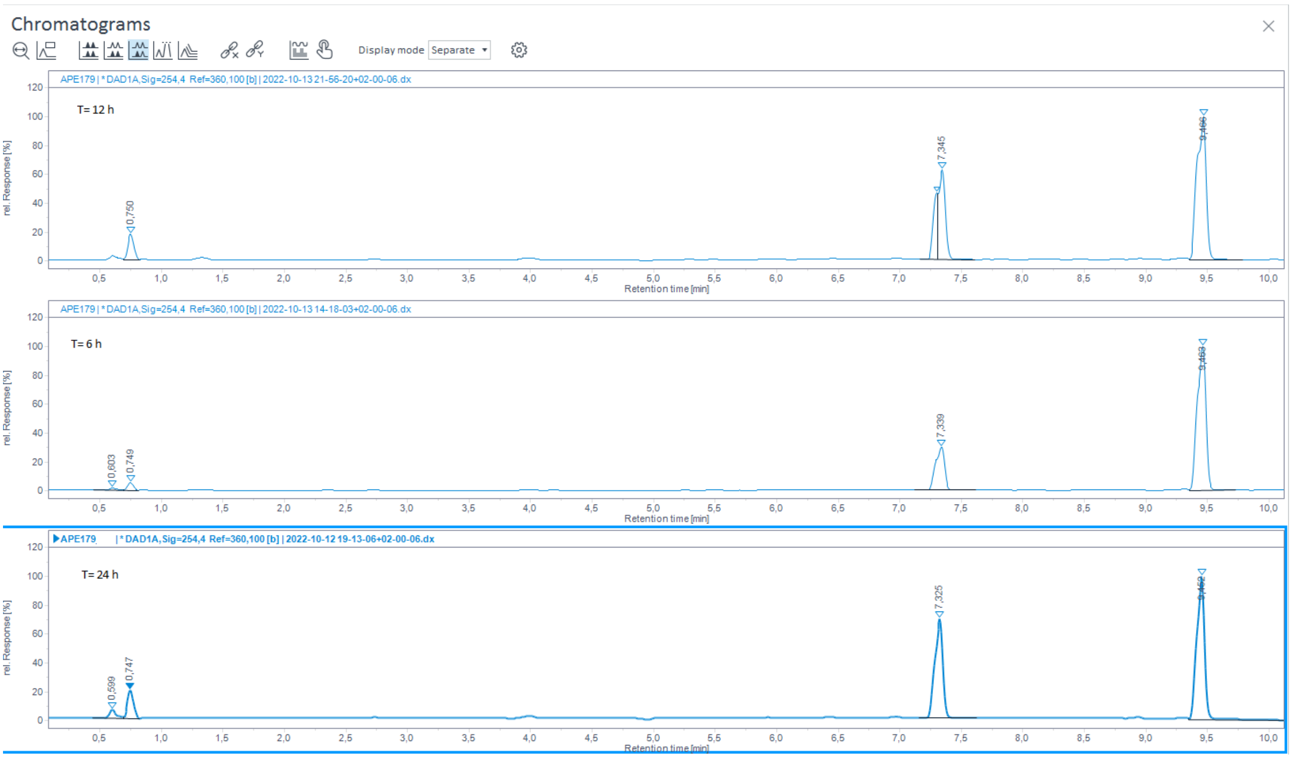

Figure S21: Hydrolysis of compound **16** at different times (6 h,12 h and 24 h)

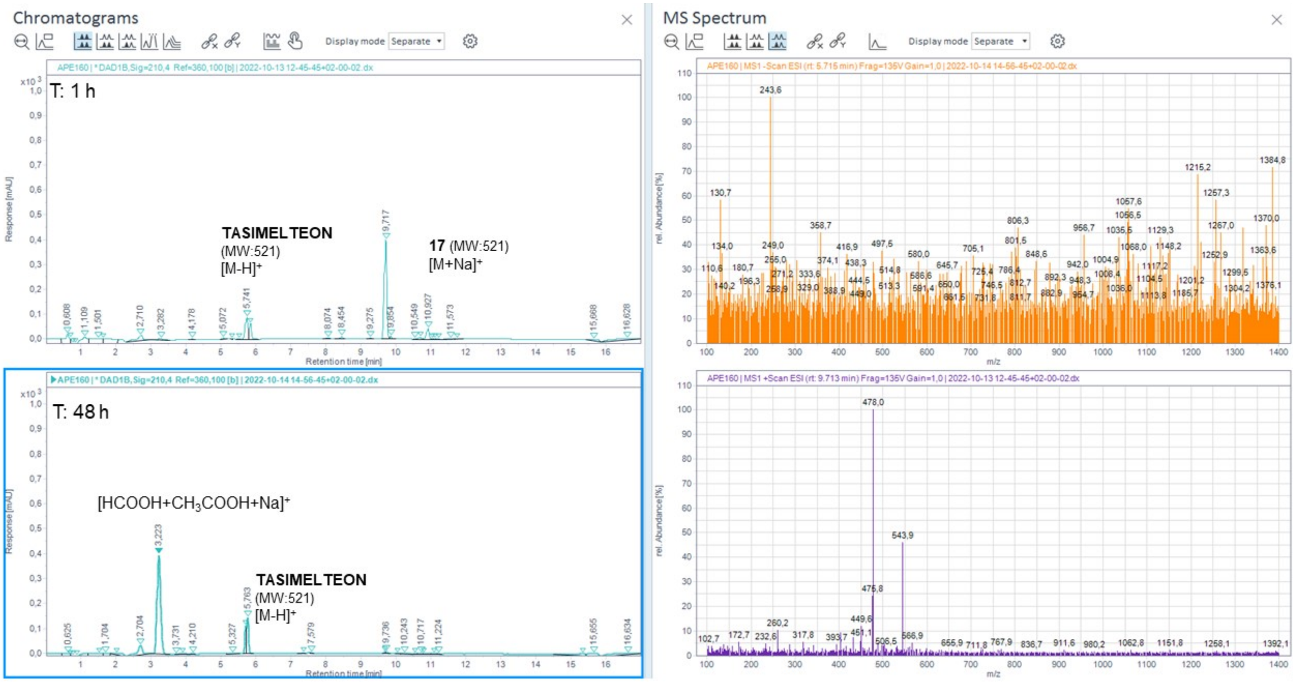

Figure S22: Hydrolysis of compound **17** at pH 5.5. Time 1 h and 48 h

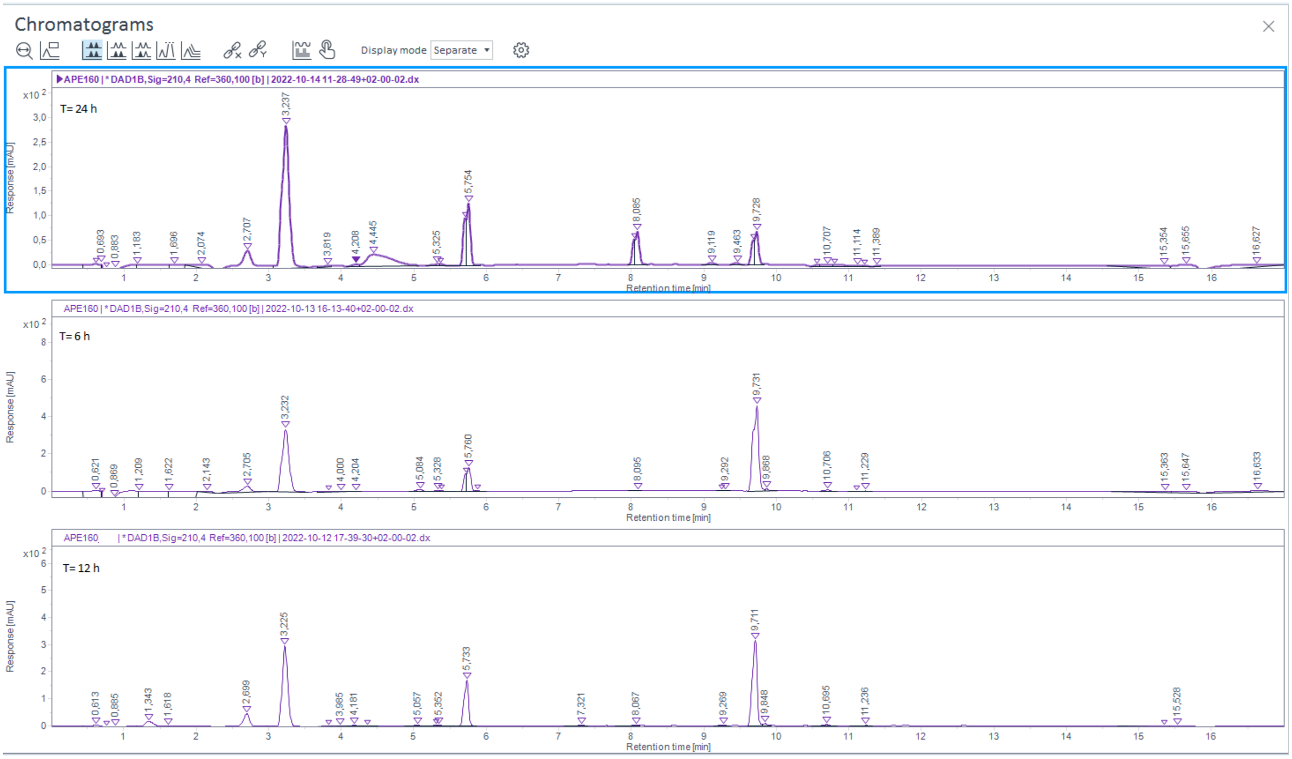

Figure S23: comparison hydrolysis of compound **17** at different time (6 h,12 h and 24 h)

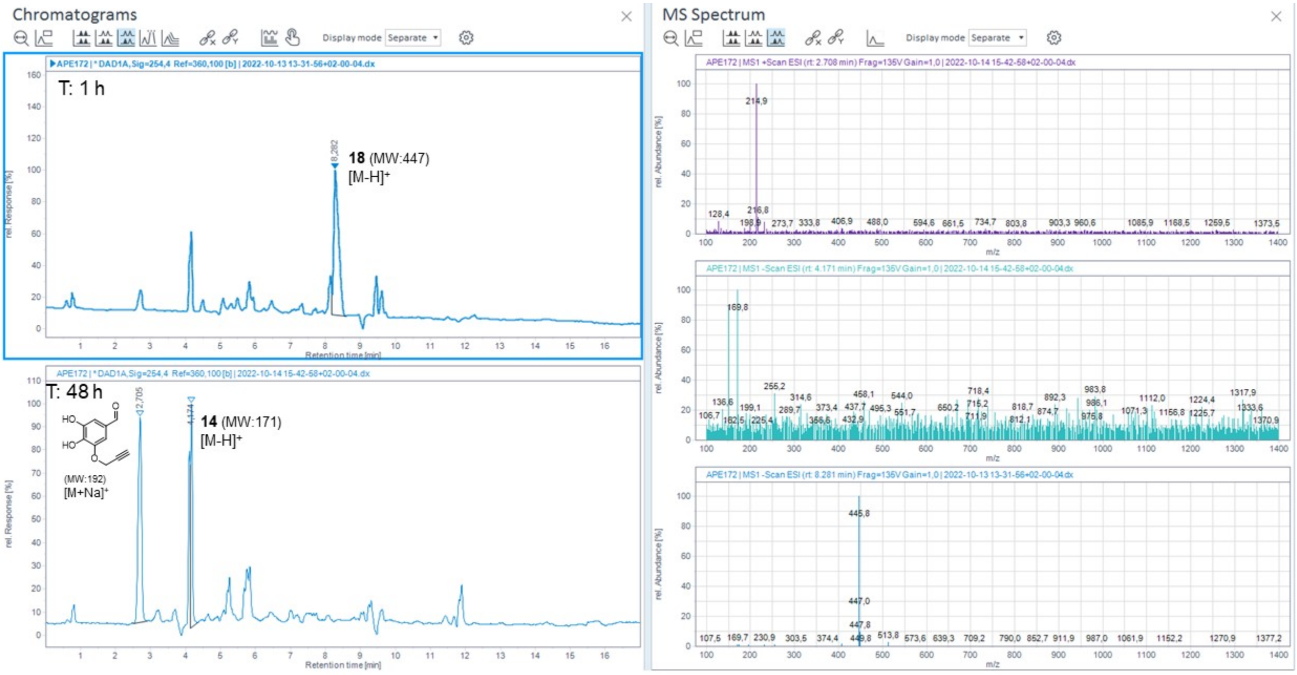

Figure S24: Hydrolysis of compound **18** at pH 5.5. Time 1 h and 48 h

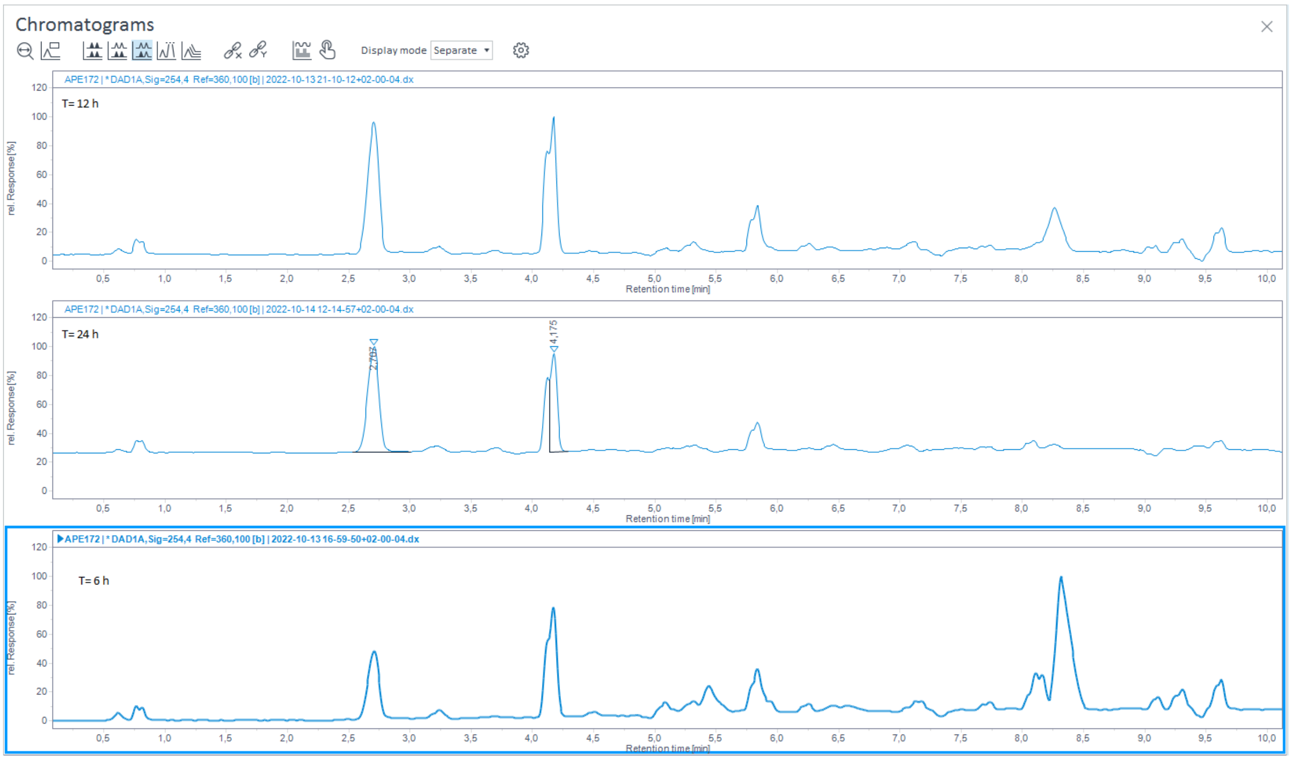

Figure S25: Comparison hydrolysis of compound **18** at different time (6 h,12 h and 24 h)

HLPC spectra (Figure S26-S29)

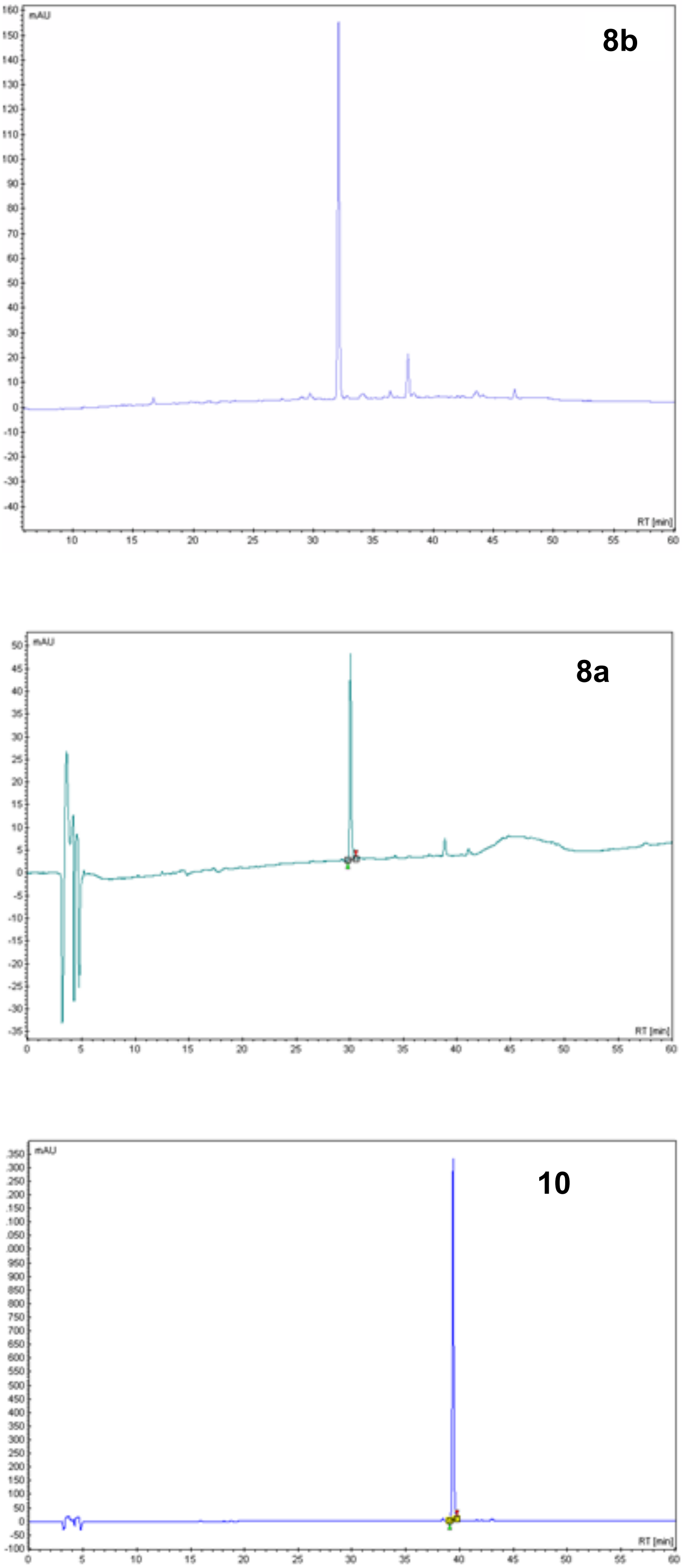

Figure S26: HPLC of pure compounds **8b**, **8a**, **10.**

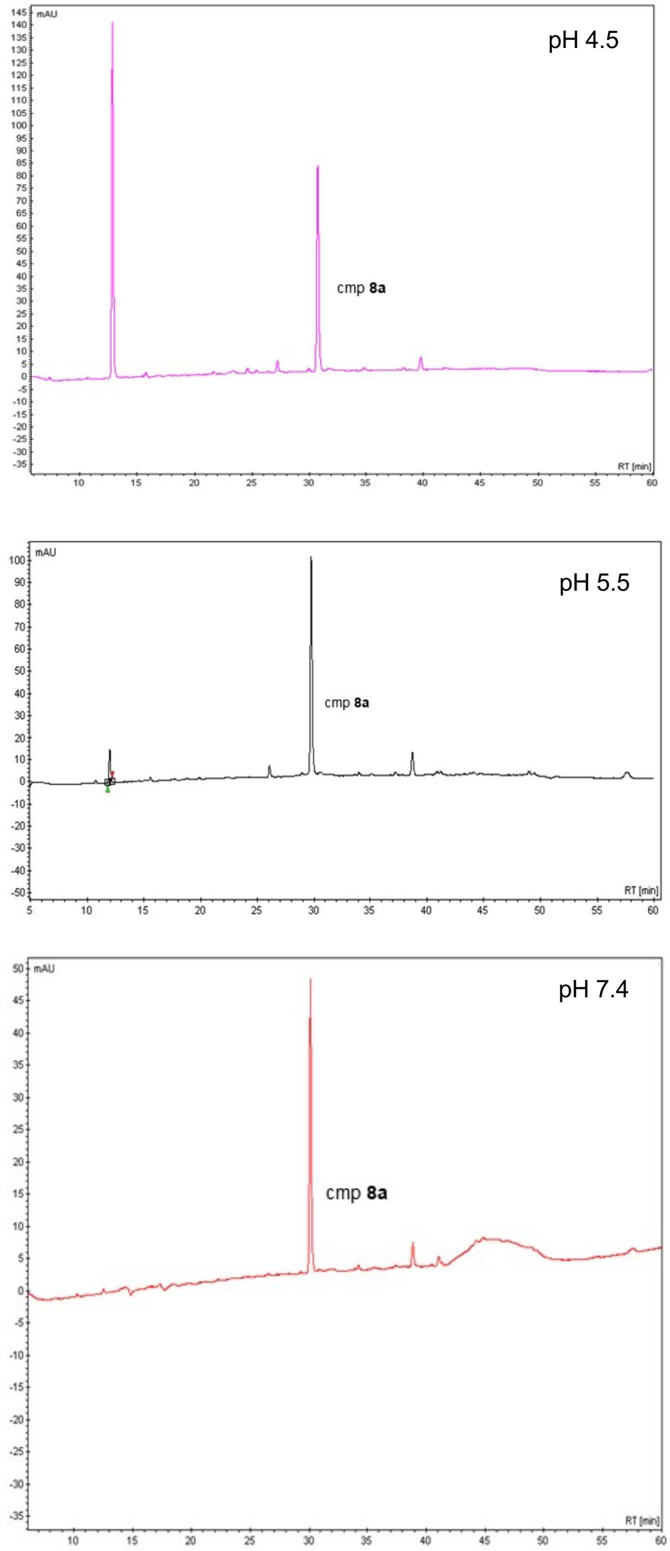

Figure S27: Behaviour of compound **8a** after 48 h at different pH values (4.5; 5.5; 7.4).

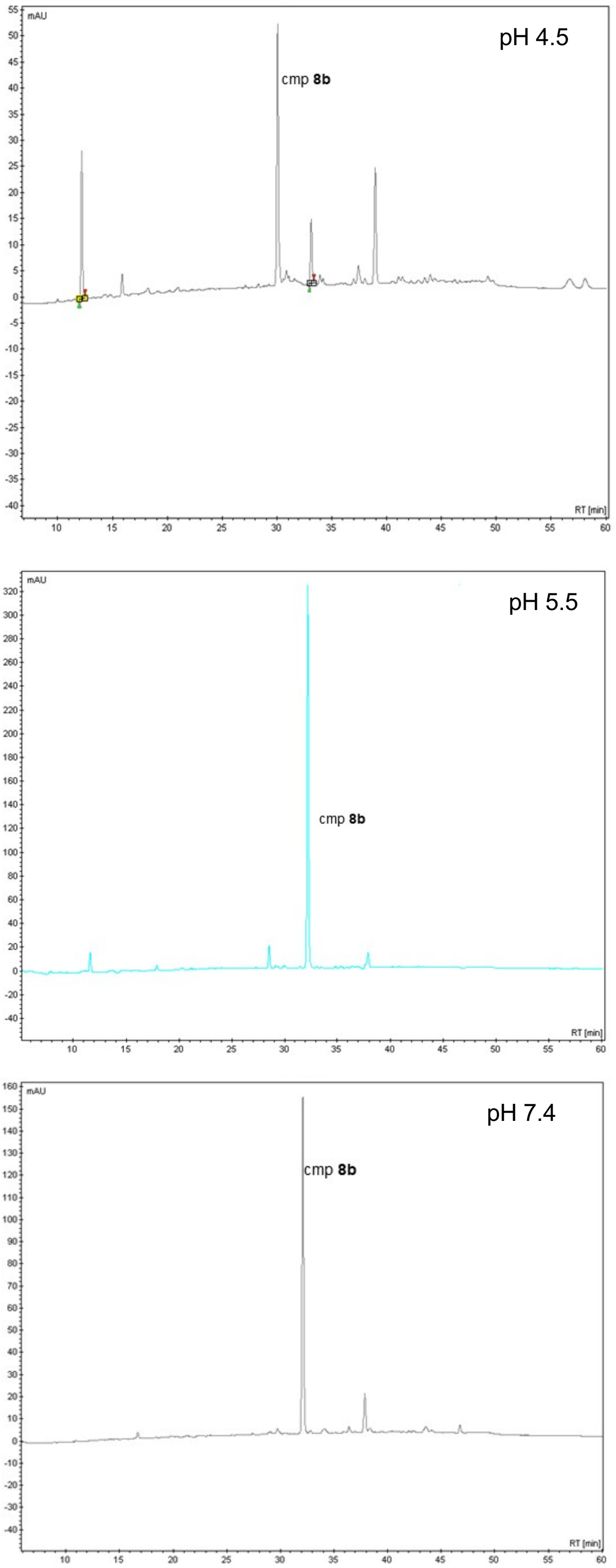

Figure S28: Behaviour of compound **8b** after 48 h at different pH values (4.5; 5.5; 7.4).

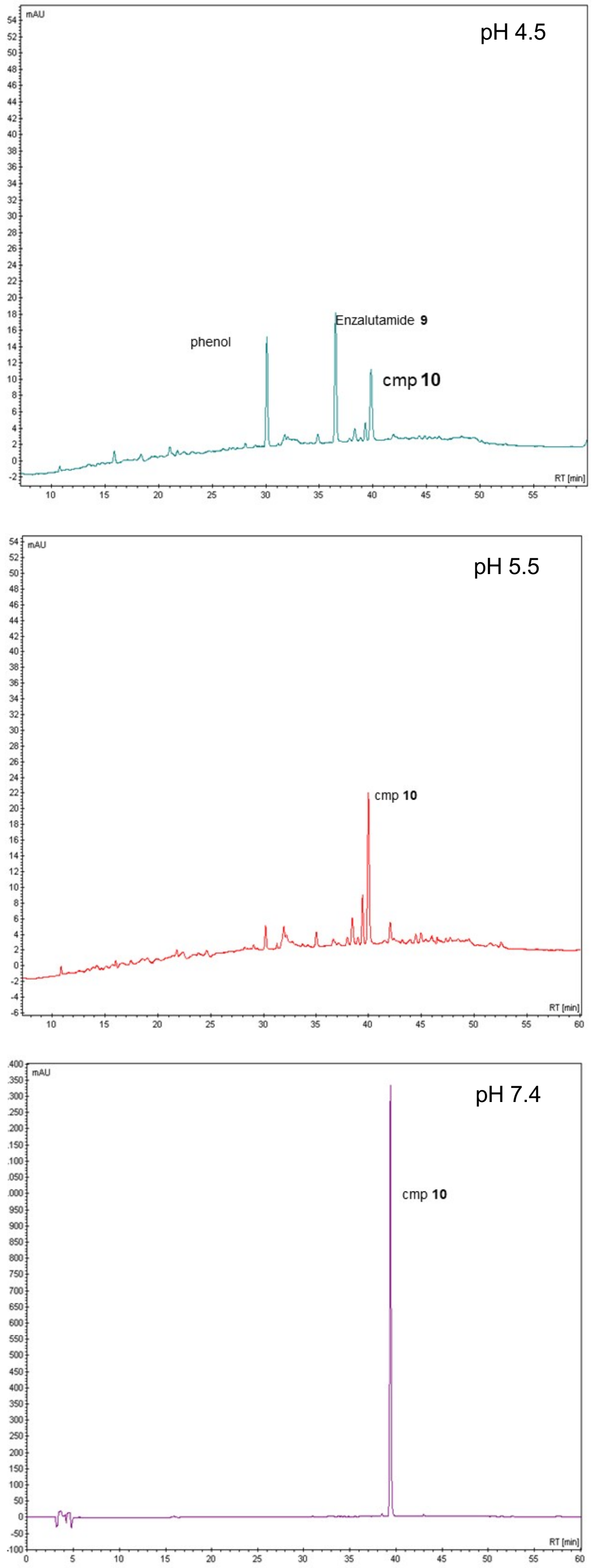

Figure S29: Behaviour of compound **10** after 48 h at different pH values (4.5; 5.5; 7.4).

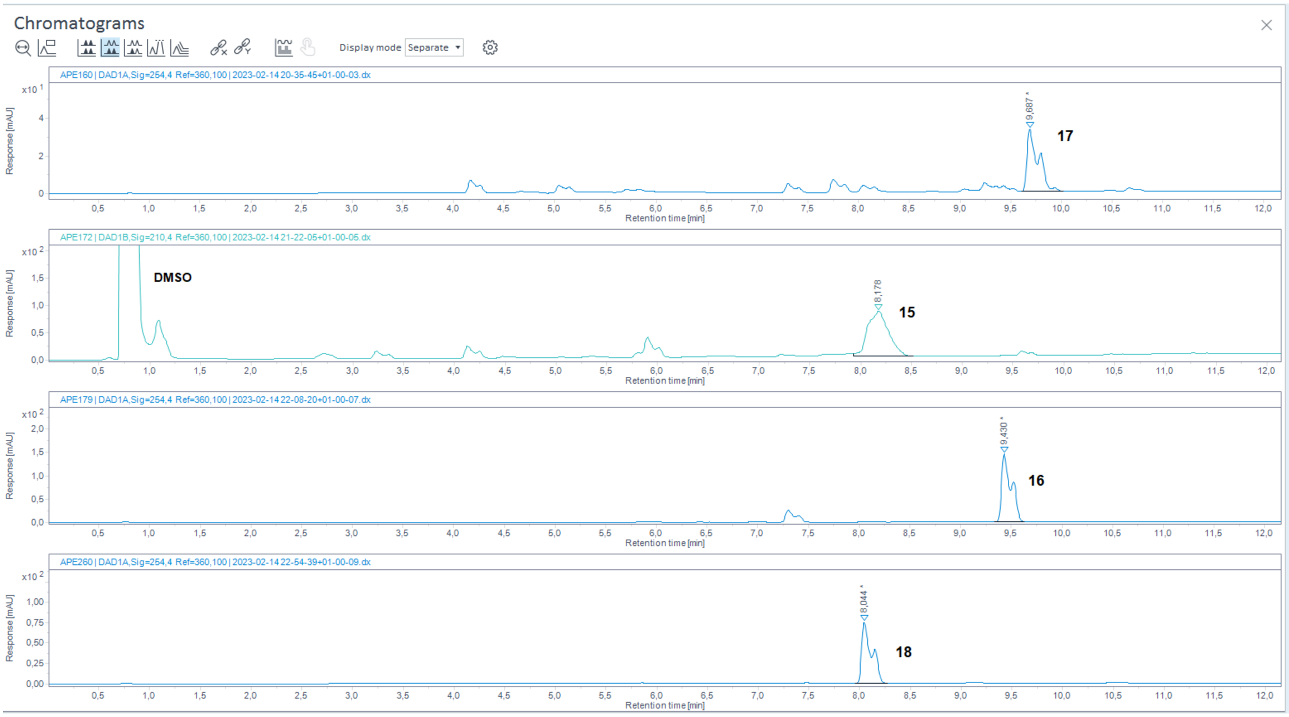

Figure S30: HPLC spectra of extracted products after 36 h in human plasma.
